# Open–closed switching of synthetic tubular pores

**DOI:** 10.1038/ncomms9650

**Published:** 2015-10-12

**Authors:** Yongju Kim, Jiheong Kang, Bowen Shen, Yanqiu Wang, Ying He, Myongsoo Lee

**Affiliations:** 1 State Key Laboratory for Supramolecular Structure and Materials, College of Chemistry, Jilin University, Changchun 130012, China; 2 Department of Chemistry and Biotechnology, School of Engineering, The University of Tokyo, 7-3-1 Hongo, Bunkyo-ku, Tokyo 113-8656, Japan

## Abstract

While encouraging progress has been made on switchable nanopores to mimic biological channels and pores, it remains a great challenge to realize long tubular pores with a dynamic open–closed motion. Here we report μm-long, dynamic tubular pores that undergo rapid switching between open and closed states in response to a thermal signal in water. The tubular walls consist of laterally associated primary fibrils stacked from disc-shaped molecules in which the discs readily tilt by means of thermally regulated dehydration of the oligoether chains placed on the wall surfaces. Notably, this pore switching mediates a controlled water-pumping catalytic action for the dehydrative cyclization of adenosine monophosphate to produce metabolically active cyclic adenosine monophosphate. We believe that our work may allow the creation of a variety of dynamic pore structures with complex functions arising from open–closed motion.

## Abstract (web summary)

Synthetic switchable nanopores may be useful for mimicking biological processes. Here, the authors report dynamic tubular pores that undergo rapid switching between open and closed states in response to a thermal signal in water and which can mediate a controlled water-pumping catalytic action.

## Graphical abstract

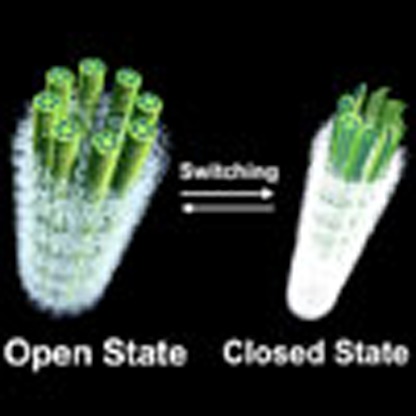

Protein pores with open–closed motion lead to various vital processes such as ion pumping, signal transduction and metabolic activites^1^,^2^,^3^. A typical example is provided by proteasome that is a hollow protein complex playing an important role in cellular function by degrading protein substrates that no longer are required or that have become damaged^4^,^5^. Regulation of proteasome activity occurs through open–closed gating motion of pores at the top and bottom ends of the symmetric proteasome barrel and restricting access to catalytic sites sequestered in the lumen of the structure. Protein pores of microtubules are also highly dynamic, continuously switching between assembly and disassembly, which initiates many important cellular functions such as organization of intracellular structure and intracellular transport, as well as ciliary and flagellar motility through reversible polymerization in response to cellular signals^6^,^7^. Inspired by such natural systems, a challenging target is how to confer switching functions with synthetic pore assemblies^8^,^9^,^10^,^11^. In fact, the synthetic tubular pores are far from dynamic switching between open and closed states^12^,^13^.

Here we report μm-long, highly dynamic tubular pores that undergo rapid switching between open and closed states triggered by a thermal signal in water. The tubular walls consist of laterally associated primary fibrils stacked from disc-shaped molecules in which the discs readily tilt by means of thermally regulated dehydration of the oligoether chains placed on the wall surfaces. Notably, the pore switching mediates a controlled water-pumping catalytic action for the dehydrative cyclization of AMP to produce metabolically active cyclic AMP (cAMP).

## Results

### Self-assembled synthetic tubule

**Figure 1:**
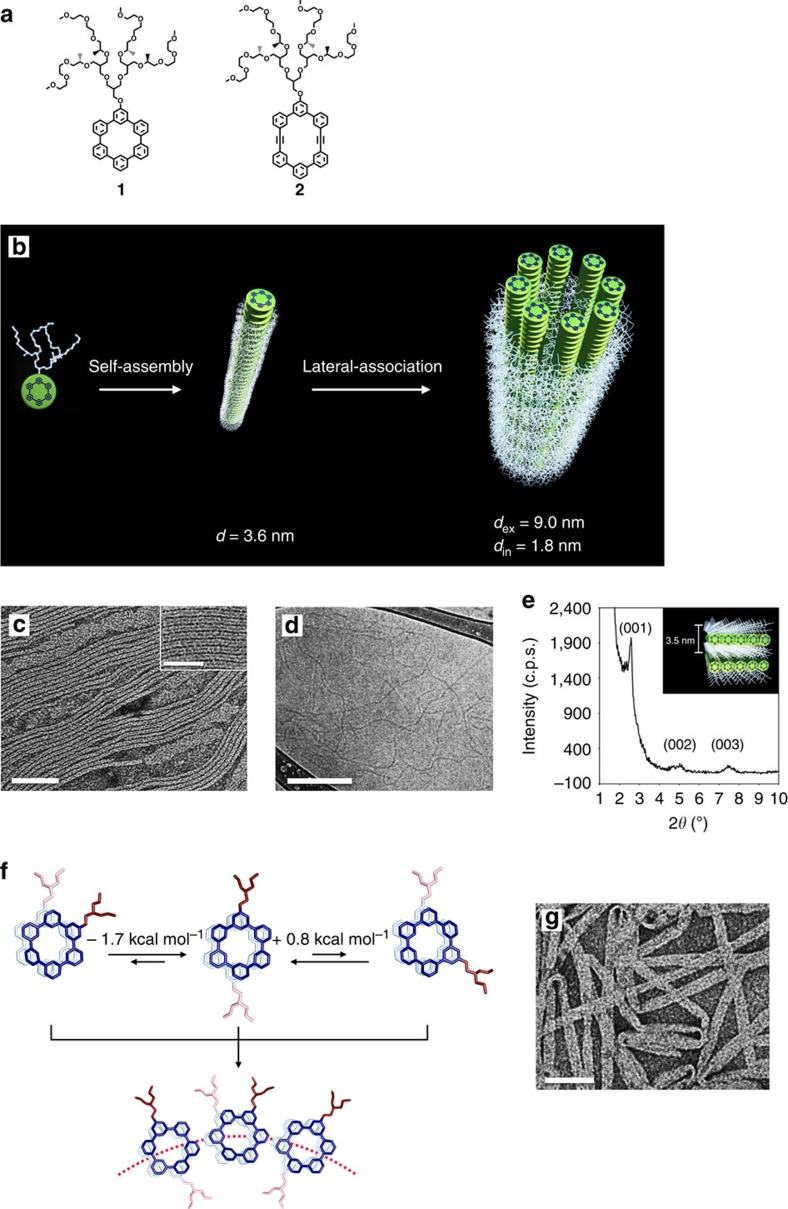
Formation of tubular nanostructure from disc amphiphile 1. (**a**) Molecular structure of macrocyclic disc amphiphiles with and a hydrophilic oligoether dendron at the periphery. (**b**) Schematic representation of fibre and tubular structures. (**c**) Negative-stain TEM image. Scale bar, 100 nm. Inset is magnified TEM image; inset scale bar, 50 nm. (**d**) Cryo-TEM image of **1** from 0.01 wt% aqueous solution. Scale bar, 200 nm. (**e**) X-ray diffraction pattern on the film from evaporation of aqueous solution of **1** at room temperature (ca. 25 °C). (**f**) DFT calculation. The energy calculation of dimers with rotation on the aromatic disc shows the low energy barrier of the mutual rotation of stacked aromatic discs, suggesting the curvature to form tubular structure from fibres. (**f**) Negative-stain TEM image of flat ribbon structures of **2** from 0.01 wt% aqueous solution. Scale bar, 100 nm. c.p.s., counts per second.

The realization of the complexity of protein assemblies in nature requires to create dynamic nanostructures with controllable functions. Self-assembly of aromatic building blocks provides a fascile means to construct dynamic nanostructures with responsive functions^14^,^15^. In contrast to the self-assembling systems based on hydrogen bonds or coordination interactions that are not highly compatible with a dynamic structural change without bond breaking^12^,^13^, nonspecific aromatic interactions allow neighbouring aromatic segments to readily rearrange with each other in response to environmental changes, leading to a switchable nanostructure without structural collapse^16^,^17^,^18^. By using the self-assembly of disc-shaped aromatic macrocycle amphiphile **1**, we have developed highly dynamic tubular pores that undergo rapid switching between open and closed states triggered by a thermal signal in water (Fig. 1). The pore walls are composed of the lateral arrangements of the thin primary fibrils formed from the stacking of disc-shaped aromatic macrocycles in which the disc stacks are able to readily tilt with respect to the normal of the fibre axis on heating^19^. The aromatic discs stacked into the primary fibrils consist of cyclohexa-*m*-phenylene and a hydrophilic oligoether dendron at the periphery (Fig. 1). To control the lateral association of the primary fibrils and endow the molecules with water-mediated assembling nature, we have incorporated a hydrophilic oligoether dendron into the aromatic periphery.

The amphiphilic molecules based on a macrocyclic disc were synthesized from commercially available starting materials according to the procedures reported previously (Supplementary Methods and Supplementary Figs 21 and 22)^20^. The disc amphiphile forms hollow tubules, as evidenced by transmission electron microscopy (TEM). When the samples were cast from the aqueous solutions of **1** (0.01 wt%), the images stained with uranyl acetate showed tubular objects with a uniform diameter of 9.0 nm that are characterized by a dark interior separated by a white periphery, indicating that **1** forms tubular pores (Fig. 1c and Supplementary Fig. 17). The formation of tubules in bulk solution was also confirmed using cryogenic-TEM (cryo-TEM) with the vitrified solutions which revealed μm-long fibre-like structures with a uniform diameter of 6.6 nm (Fig. 1d). This mismatch in diameter originates from solvated ethylene oxide chains that do not provide enough contrast for direct observations^21^, supporting that the external thickness of the oligoether part is 1.2 nm. Considering the macrocycle diameter of 1.2 nm, the tubular wall is estimated to be 3.6-nm thick, resulting in an internal pore diameter of 1.8 nm. The wall thickness is in reasonable agreement with the bilayer thickness (3.5 nm) determined from X-ray diffraction performed on the films prepared from the evaporation of the aqueous solution (Fig. 1e and Supplementary Fig. 15). To understand the formation mechanism of the tubular walls, we additionally performed TEM experiments at a highly diluted condition (0.003 wt%) of **1** (Supplementary Fig. 1). The image showed thin fibrils with an external diameter of 3.6 nm, demonstrating that the tubular walls are composed of the lateral association of the primary fibrils formed at initial self-assembly conditions (Fig. 1b). Considering the measured tubular diameter of 9.0 nm and the fibril diameter of 3.6 nm, the number of the elementary fibrils consisting of a tubular wall is estimated to be 8.

According to the molecular modelling, the aromatic scaffolds of the primary fibrils are not entirely surrounded by hydrophilic chains. To reduce the contact between aromatic segments and water molecules, the primary fibrils laterally assemble into tubular structures at higher concentrations. This is further supported by the formation of stable fibrils from the self-assembly of the macrocycle amphiphile with longer hydrophilic chains (Supplementary Fig. 2). We observed that the formed fibrils (∼5 nm in diameter) are stable without further aggregation even at higher concentrations. It is worthy of noting that the lateral interactions of aromatic fibres consisting of anisotropic rods give rise to flat ribbon structures based on symmetric layer packings^22^. However, the aromatic discs of **1** are able to readily rotate with respect to the neighbouring discs to form asymmetric bilayer packings^20^, as illustrated by density functional theory (DFT) energy calculations (Fig. 1f). The asymmetric placements of hydrophilic chains between the basal planes caused by the mutual rotation of stacked aromatic discs would drive the lateral association of the fibrils to be curved to meet space-filling requirements for the flexible chains. To support this explanation, we prepared macrocycle amphiphile **2** based on an elliptical disc, which has higher rotational energy barrier than **1** for the mutual rotation between the adjacent molecules (Supplementary Fig. 3). Indeed, **2** forms a flat ribbon structure with a width of ∼30 nm consisting of the lateral assembly of primary fibrils (Fig. 1g), further supporting that the curvature of the tubular walls originates from the asymmetric locations of hydrophilic dendritic chains between up and down the basal planes^23^.

### Open–closed switching of tubular pores

**Figure 2:**
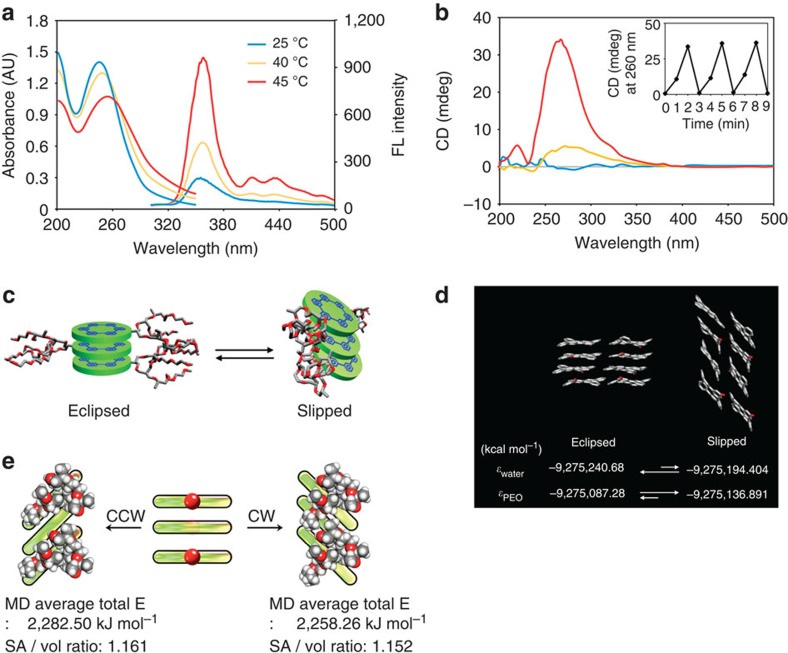
Thermoresponsive properties of self-assembled tubules from disc amphiphile 1. (**a**) Temperature-dependent absorption and emission spectra and (**b**) circular dichroism (CD) spectra of **1** from 0.03 wt% aqueous solution. The inset shows the reversible induction of CD signal as to the heating/cooling cycles. (**c**) Schematic representation of the switching motion between eclipsed and slipped packing arrangement of the disc planes. (**d**) Prediction of thermophysical properties of eclipsed and slipped stacking based on octamer between room temperature and LCST. We presumed that octamer might be surrounded by water in room temperature or PEO over LCST. (**e**) Molecular dynamics (MD) simulations between clockwise (CW) tilting and CCW tilting. MD average total energy shows that CW tilting direction is preferred due to more compact packing (lower surface area/volume (SA/vol) ratio) in oligoether dendron.

The tubules of **1** exhibit thermoresponsive properties due to thermally regulated dehydration of the ethylene oxide chains on the tubular walls^24^. The temperature-dependent transmittance of the aqueous solutions of **1** showed a sharp phase transition at about 40 °C (Supplementary Fig. 4), indicating that the ethylene oxide chains with open conformations are dehydrated to collapse into a globular conformation on heating. The conformational change of the oligoether chains would influence the packing mode of the macrocycle stackings due to an increased cross-sectional area of the globules and strengthened hydrophobic interactions. This is reflected in the red-shifted absorption maximum and fluorescence enhancement on heating (Fig. 2a). We believe that the observed spectral changes are the result of a slipped packing arrangement of the disc planes^25^. At room temperature, the macrocyclic discs stack perpendicular to the fibre axis to maximize aromatic interactions. On heating, however, the dehydrated oligoether globules would make the eclipsed stackings of the discs to be unstable due to steric crowding between the dehydrated globules with a greater cross-section. To relieve the steric crowding at the interface, the disc planes would tilt relative to the normal of fibre axis through the sliding with respect to each other to allow a larger interfacial area (Fig. 2c), thus lowering total free energy^17^. Computational molecular simulations of **1** based on quantum chemical (DFT/COSMO (COnductor-like Screening MOdel)) calculations supported attribution of the slipped packing to dehydration of the dendritic chains on the tubular walls. According to the calculation, the tilted packings based on a slipped arrangement of the aromatic discs are more favourable than the eclipsed packings in a dehydrated environment, as opposed to that in a hydrophilic medium (Fig. 2d). This result is in good agreement with the spectral changes in the dehydrated state of the oligoether chains. Interestingly, the molecular tilting is accompanied by the reversible induction of a strong circular dichroism signal as to the heating/cooling cycles (Fig. 2b). The circular dichroism signal appears immediately in response to even fast heating within a few minutes, indicating that the tilting undergoes rapid with a preferred direction. Assemblies derived from the enantiomer of **1** exhibit opposite circular dichroism signals with a mirror image relationship, indicating that the molecular chirality is transferred to the self-assembled structure (Supplementary Fig. 5). To corroborate the preferred tilting direction triggered by the chiral oligoether chains, we performed molecular dynamics simulations to compare the average energy for flexible oligoether chain conformation between clockwise tilting and counterclockwise (CCW) tilting. According to the calculation, clockwise tilting allows the anisotropic chiral oligoether chains to adopt lower average energy and more compact packing (lower surface area/volume ratio) compared with those of CCW tilting (Fig. 2e and Supplementary Fig. 16), rendering chirality with the tubules.

**Figure 3:**
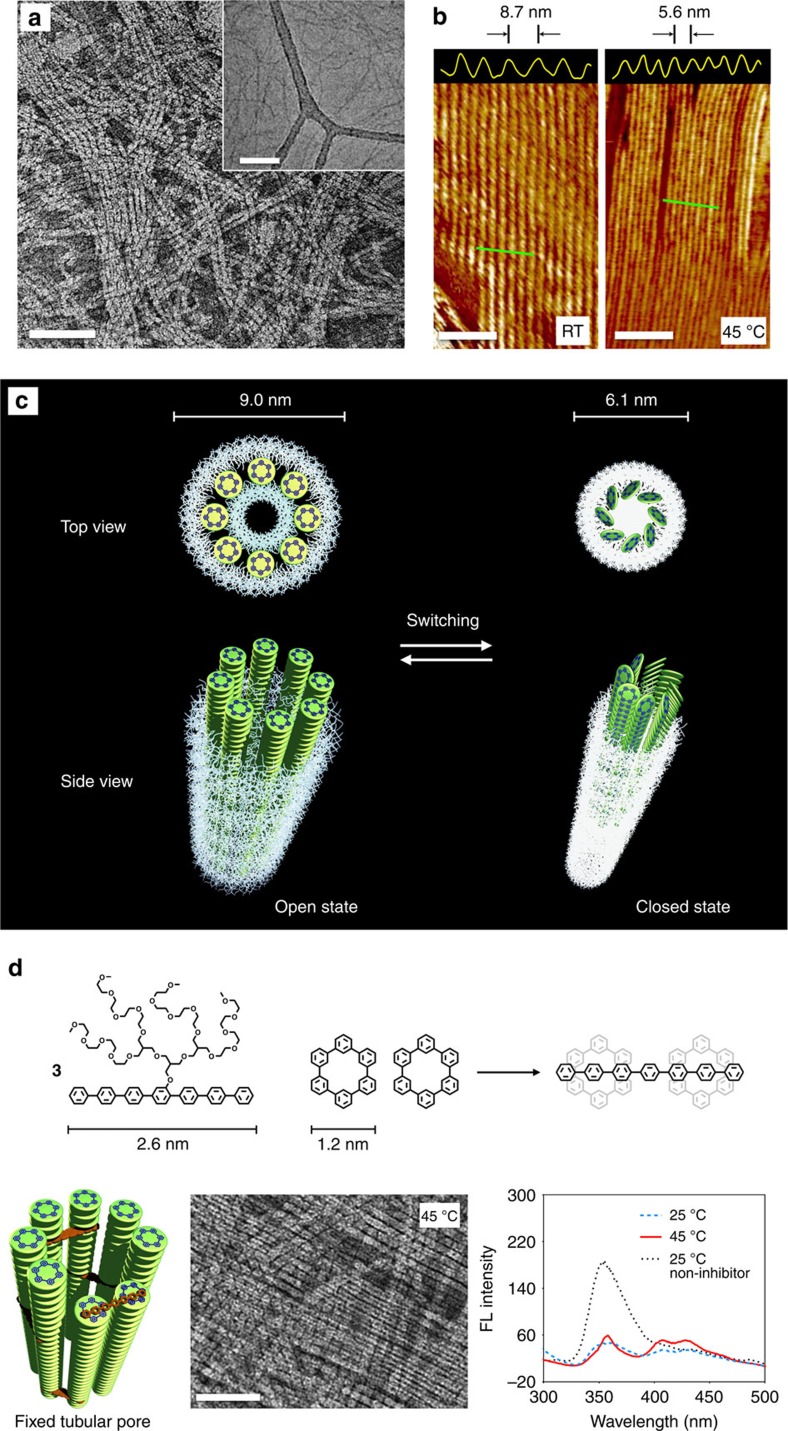
Open–closed switching of tubular pores. (**a**) Negative-stain TEM image and cryo-TEM image (inset) of **1** from 0.01 wt% aqueous solution prepared at 45 °C. Scale bars, 50 nm. (**b**) AFM phase images of the films from evaporation of aqueous solution of **1** from 0.01 wt% aqueous solution prepared at 25 and 45 °C. The tubular shrinkage was confirmed by the reduction of the diameter in closely packed fibre-like aggregates from 8.7 to 5.6 nm. Scale bars, 50 nm. (**c**) Schematic representation of the open–closed switching of the tubular pores with the slipped packing arrangements of the macrocyclic discs. (**d**) Inhibition of the tilt of the disc planes and pore closing. Amphiphile **3** as inhibitor prevents the tilt of the disc planes by the intercalation between the disc stacking. The dark interior of tubules were preserved even after heating in negative-stain TEM image. Emission spectra of **1** from 0.01 wt% aqueous solution in the presence of 0.0005, wt% amphiphile **3** show fluorescence quenching of **1**. Scale bar, 50 nm. RT, room temperature.

The tilting of the macrocyclic discs leads to drastic shrinkage of the tubules in cross-section, which was directly visualized by TEM. On heating the solution of **1**, the image showed fibre-like structures with a diameter of 6.1 nm (Fig. 3a), indicative of a 32% reduction in external diameter of the tubule with respect to that at room temperature. The shrinkage in bulk solution was also confirmed by cryo-TEM, which showed fibre-like structures with a diameter of 3.5 nm (Fig. 3a, inset and Supplementary Fig. 18), demonstrating a significant reduction in the tubular cross-section on heating. Closer examinations of the stained samples revealed that the shrunken tubules appear to be a lack of dark interior associated with hollow space, demonstrating that the thermally regulated shrinkage of the tubules makes the internal pores to be closed. Closing the tubular pores on heating is also manifested in even larger reduction (2.9 nm) in external diameter of the tubules than the 1.8-nm pore size. This additional reduction in external diameter arises from the increase in the interfacial area to the fibre axis direction caused by the tilt of the disc planes^19^. The tubular shrinkage was also confirmed by atomic force microscopy (AFM) measurements on hydrophilic mica substrates in the completely dried state (Fig. 3b and Supplementary Fig. 14). The image derived from the eclipsed stackings revealed closely packed fibre-like aggregates with a diameter of 8.7 nm, while the tilted stackings (slipped packings) revealed the fibres to be shrunken to 5.6 nm in diameter. This reduction in diameter is in good agreement with the results obtained from TEM. The pore closing, together with all the spectral changes, is fully reversible on cooling and subsequent heating cycles.

Taken all the data together, we propose the mechanism of the open–closed switching of the tubular pores considering the slipped packing arrangements of the macrocyclic discs at the structural transition (Fig. 3c). On heating, the eclipsed stackings of the macrocyclic discs in the primary fibrils are tilted with a clockwise direction with respect to the normal of the fibre axis through the sliding of disc planes. The tilting of the aromatic discs drives a significant reduction in the cross-section of the primary fibrils, which enforces the tubular pores to be closed to preserve the lateral interactions between the primary fibrils. To further support this explanation, we prepared rod amphiphile **3** based on hepta-*p*-phenylene to be able to fix the pore structures through preventing the tilt of the disc planes (Fig. 3d), because the amphiphilic rods with a length of 2.6 nm would be intercalated between the disc stackings to build a bridge between the adjacent primary fibrils. The intercalation of **3** into the tubular walls was observed, which was confirmed by fluorescence quenching of **1**. Indeed, on heating the mixture solutions of **1** containing 5 wt % of **3**, the fluorescence emission remained unchanged with a lack of circular dichroism signals (Fig. 3d and Supplementary Fig. 6). Furthermore, the dark interiors of the tubules of the stained sample in the TEM image were preserved even after heating (Fig. 3d). These results demonstrate that **3** functions as an effective inhibitor to prevent the tilt of the macrocyclic discs and thus, fix the pore structures.

### Semipermeable tubular wall

Similar to a semipermeable biological membrane, small water molecules can readily pass through across the tubular walls through free diffusion because the solution self-assembly leads the walls to be based on dynamic empty spaces not only between the disc stackings but also between the laterally associated primary fibrils. Furthermore, closing the tubular pores is believed to highly accelerate squeezing out water from the internal pores across the walls as well as through the open ends. This is manifested by the rapid switching between open and closed states, which strongly enforces water to squeeze out of the internal pores. To corroborate the semipermeable nature of our tubular walls, we encapsulated a series of guest molecules into tubular pore (Supplementary Fig. 7). After 1 h at 45 °C, releasing percentage of the encapsulated guest molecules shows that the larger molecule was released out more slowly, implying the size dependency of permeable guest molecules across the tubular wall. In addition, the release rate of the encapsulated molecule within the tubular pores was obtained using calcein dye (Supplementary Fig. 8). On heating the solution to 45 °C, at which the internal pores are closed, the release of encapsulated calcein was monitored by a decrease in absorbance in 491 nm and an increase in fluorescence emission in 515 nm as the free calcein in solution was dequenched^26^. At the initial stage up to 1 h, essentially no leakage of the encapsulated calcein was observed, and then slowly released from the closed state of the tubular pores over 12 h, while the calcein was released out of the tubular pores over a period of 5 days in the open state at room temperature (Supplementary Fig. 9). The results, together with fast open–closed switching of the tubular pores, indicate that the tubular walls function as a semipermeable membrane for the rapid squeezing out water from the internal pores while entrapping the larger dye molecules over a period of several hours within a confined, hydrophobic environment driven by the dehydration-mediated pore closing.

### Dehydration reaction of AMP

**Figure 4:**
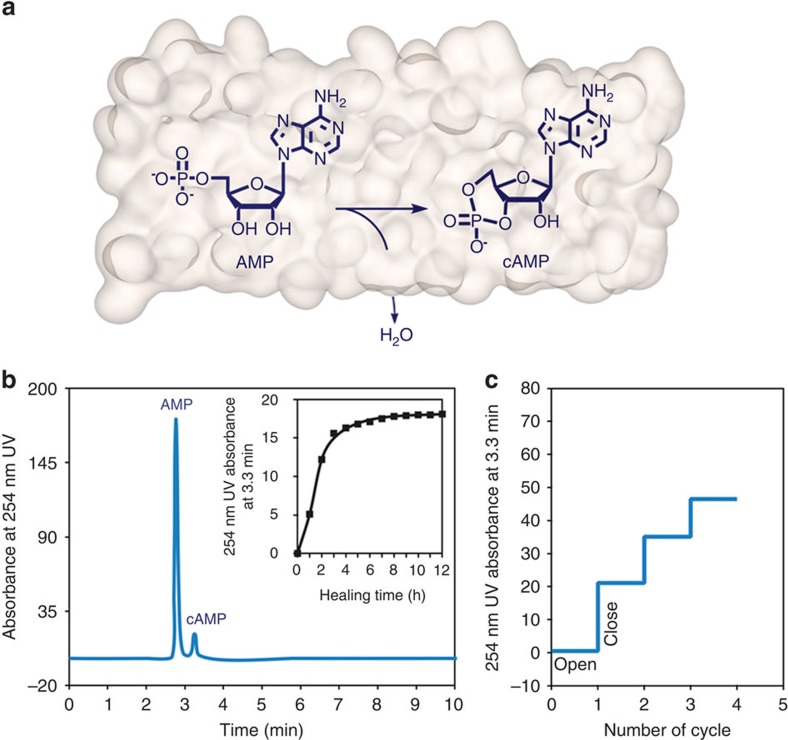
Dehydrative cyclization of AMP. (**a**) Schematic representation of dehydrative cyclization of AMP through the closed state of the tubular membranes. (**b**) HPLC spectra of dehydration result in the closed state after separation of the free AMP from the mixture solution of the tubules. The inset shows that the peak intensity at 3.3 min increases gradually over 3 h. (**c**) In the presence of free AMP without separation, the open–closed switching cycles of the tubular pores increase the amount of the cyclized product.

Accordingly, we considered that the open/closed switching of the tubular pores could be utilized for the abiotic dehydration of encapsulated biomolecules in aqueous media (Fig. 4a)^27^. As a proof of this idea, we encapsulated AMP into the tubular pores to perform a dehydrative cyclization to produce cAMP, which is an important second messenger that mediates intracellular signal transduction in many different organisms^28^. *In vivo*, cAMP is formed from ATP by the enzymatic action of adenyl cyclase located on the inner side of the plasma membrane. The intracellular actions of cAMP are terminated by the enzymatic hydrolysis by phosphodiesterase to form inactive AMP. However, the reverse reaction of AMP to produce active cAMP remains elusive. Furthermore, abiotic dehydrative cyclization of AMP is essentially limited in water because it requires not only high energy for the cyclic bond formation but also the removal of water produced in the reaction^29^. To address this challenge, we carried out the dehydrative cyclization of AMP using our tubular membranes in their closed state (Fig. 4a). The encapsulation of AMP within the tubular pores (10 mM) was confirmed by tracing high-performance liquid chromatography (HPLC) after separation of the free AMP from the mixture solution of the tubules and AMP using a Sephadex column (Supplementary Figs 10 and 12). On heating, an additional peak corresponding to cAMP was identified in analytical HPLC of which the intensity increases gradually over 3 h, demonstrating that the dehydrative cyclization undergoes successful in water media (Fig. 4b). In the presence of free AMP before separation, the reaction could be regulated through the open–closed switching cycles of the tubular pores (Fig. 4c and Supplementary Fig. 11). With increasing the number of subsequent cooling and heating cycles, the amount of the cyclized product increases consecutively, indicating that free AMP is diffused into the tubular pores in the open state and then the reaction occurs repeatedly in the closed state. The dehydration reaction yield of AMP in bulk sample solution was obtained as 1.065 × 10^−4^% per cycle (Supplementary Fig. 19). Moreover, the water-pumping catalytic activity of our tubules could be readily inhibited by the addition of rod amphiphile **3** that fix the pore structures. Indeed, we were not able to observe any noticeable products when the tubular solution included 5 wt % of **3**, indicating that the water-pumping catalytic activity of the tubular pores is controlled with an aid of the inhibitor molecule. The driving forces responsible for the dehydrative cyclization are believed to be the space confinement and hydrophobic environment generated by dehydration of the oligoether chains located at the inner pore walls. The dehydration was confirmed by temperature variable nuclear magnetic resonance (NMR) experiments, which show upfield shifted NMR peaks of the oligoether dendrons together with peak broadening (Supplementary Fig. 20). The space confinement would considerably restrict rotational degrees of freedom during the cyclization of the open form, which lowers the activation energy of the ring-closure reaction^27^. The facile dehydration reaction attributed to the hydrophobic environment of the inner pore was also confirmed by an imine formation reaction (Supplementary Fig. 13), further supporting that space confinement together with the hydrophobic environment through the water-pumping action of the tubular pores is a major driving force for the dehydration reactions in water.

## Discussion

We realized highly dynamic tubular pores that are rapidly switching between open and closed states triggered by a thermal signal, giving rise to a unique water-pumping catalytic action to create a metabolically active biomolecule from inactive AMP, by using the self-assembly of disc-shaped aromatic macrocycle amphiphile. We believe that our approach for designing new synthetic nanopores will allow the creation of a variety of switchable pore structures with complex functions arising from open–closed motion. Such a dynamic switching of the pore structures to trigger unique water-pumping action may provide new opportunities of using the hollow structures to encapsulate specific precursor biomolecules in a size-selective manner and convert them to their target molecules for the regulation of many important cellular functions through the controlled inhibition or activation of metabolic pathways.^30^,^31^

## Methods

### General

Matrix-Assisted Laser Desorption/ Ionization Time of Flight Mass Spectrometry (MALDI TOF-MS) spectroscopy was performed on a Bruker Autoflex TOF/TOF using α-cyano- 4-hydroxy cinnamic acid as a matrix. Ultraviolet/visible spectra were obtained from a Hitachi U-2900 spectrophotometer. The fluorescence spectra were obtained from a FL-5301PC (Shimadzu) fluorescence spectrophotometer. Circular dichroism spectra were obtained using Jasco J-810 spectropolarimeter. The AFM measurements were conducted on a MultiMode 8 AFM with NanoScope V controller, NanoScope software and NanoScope Analysis software (Bruker AXS Corporation, Santa Barbara, CA, USA) in air at ambient temperature (ca. 25 °C) in the tapping mode. Images were acquired in PeakForce Tapping mode. X-ray diffraction patterns were obtained using a Rigaku D/max 2550 diffractometer (Rigaku Co.). HPLC analysis was performed with Prominence LC-20AP (Shimadzu) and Vydac C18 reverse phase column (218TP54). Molecular modelling and calculations were performed by Material Studio 6.0 programme (Accelrys Software Inc.) and the MacroModel module within the molecular modelling suite Maestro from Schrödinger Suites (Schrödinger K.K.).

### TEM experiments

To investigate the structures of self-assembled structures in aqueous solution, a drop of an aqueous solution of the aromatic macrocycle amphiphiles (0.003–0.01 wt%) was placed on a carbon-coated copper grid (Carbon Type B (15–25 nm) on 200 mesh, with Formvar; Ted Pella, Inc.) and the solution was allowed to evaporate under ambient conditions. These samples were stained by depositing a drop of uranyl acetate aqueous solution (0.2–1.0 wt%) onto the surface of the sample-loaded grid. For the preparation of the heated sample, 0.01 wt % aqueous solution, carbon-coated copper grid and uranyl acetate aqueous solution were incubated in 45-°C oven for 1 h. Then, the turbid solution was cast on the grid and the solvent was evaporated in 45-°C oven for 30 min. The thin film sample was stained by 45-°C uranyl acetate aqueous solution for 1 min in 45-°C oven. The dried specimen was observed by using a JEOL-JEM HR2100 instrument operating at 160 kV. The cryo-TEM experiments were performed with a thin film of an aqueous solution of amphiphiles (5 μl) transferred to a locally supported grid in room temperature and 45 °C (Lacey Formvar/Carbon, 200 mesh, Cu, Ted Pella, Inc.). The thin aqueous films were prepared under controlled temperature and humidity conditions (97–99%) within a custom-built environmental chamber to prevent evaporation of water from sample solution. The excess liquid was blotted with filter paper for 2–3 s, and the thin aqueous films were rapidly vitrified by plunging them into liquid ethane (cooled by liquid nitrogen) at its freezing point. The grid was transferred on a Gatan 626 cryoholder using a cryo-transfer device and transferred to a JEM–HR2100–TEM. Direct imaging was carried out at a temperature of approximately −175 °C and with a 120-kV accelerating voltage, using the images acquired with a Dual vision 300 W and SC1000 CCD camera (Gatan, Inc.; Warrendale, PA). The data were analysed using Digital Micrograph software.

### AFM experiments

To prepare the heated sample, 0.01 wt% aqueous solution was incubated in 45-°C oven for 1 h. Then, 10 μl aliquots of the turbid solution were cast on mica surface and the solvent was evaporated in 45-°C oven for 30 min

### Dynamic light scattering experiments

The dynamic light scattering experiments were performed by ALV/CGS-3 Compact Goniometer System using He–Ne laser operating at 632.8 nm. The scattering was kept at 90° during the whole experiment at 25 °C. The hydrodynamic diameter (*D*_H_) was determined from the autocorrelation functions by the time interval method of photon correlation and the CONTIN methods using the software provided by the manufacturer. To avoid contamination of dust, all solutions were filtered through a 0.45-μm membrane filter.

### NMR experiment

^1^H-NMR (600 MHz) spectra were recorded using a solution of amphiphile **1** in D_2_O with a concentration of 0.06 wt%. At each temperature, the solution was equilibrated for 10 min before data acquisition. The dehydration of the ethylene oxide segments was supported by peak broadening and peak shift towards upfield^18^.

### Energy calculation

Energy of three conformations (60°, 120° and 180°) was calculated to evaluate the relative energy using Material Studio 6.0 programme (Fig. 1f). Truncated structures were used to reduce the calculation time. The preliminary geometry of the molecules was optimized through COMPASS force field of Forcite module. Then, fine geometry optimizations were performed by DMol^3^ module based on DFT under the following parameters: spin polarization: restricted; basis: dnp; pseudopotential: none; functional: pbe; aux density: octupole; occupation: Fermi; and cutoff global: 3.30 Å.

For the prediction of thermophysical properties of eclipsed/slipped stacking based on octamer (Fig. 2d) between room temperature and lower critical solution temperature (LCST), we presumed that octamer might be surrounded by water in room temperature or polyethylene oxide (PEO) in LCST. Therefore, we exploited the solvent effect between water condition and PEO condition to study the eclipsed/slipped stacking of aromatic macrocycles in below and above LCST temperature. COSMO method^32^,^33^ had been used to compare the relative energy of eclipsed/slipped octamer in different solvent condition. This is based on the approximated continuum description of solvent, in which the solute molecule forms a cavity within the dielectric continuum of permittivity, *ɛ*, that represents the solvent. We use the dielectric constant of water (*ɛ*_water_=78.5) and the dielectric constant of PEO (*ɛ*_PEO_=5.0) (ref. 34). Then, fine geometry optimizations were performed by DMol^3^ module based on DFT under the following parameters: spin polarization: restricted; basis: dnp; pseudopotential: none; functional: bp; aux density: octupole; occupation: Fermi; cutoff global: 3.70 Å; cosmo: ibs; and COSMO dielectric: 78.5 (water) or 5.0 (PEO).

The preferred tilting direction induced by the anisotropy of the chiral dendron on heating was studied by molecular dynamics simulation through MacroModel module from Schrödinger Suites (Fig. 2e). The dynamics of flexible anisotropic dendron under the constrained positions of the slipped arrangement of the aromatic discs was performed under the following parameters: force field: OPLS_2005; solvent: water; cutoff: Van der Waals (8.0)/electrostatic (20.0)/H-bond (4.0); minimization method: PRCG; maximum iterations: 2,500; converge on: gradient; convergence threshold: 0.05; dynamics method: stochastic dynamics; simulation temperature: 300.0 K; time step: 1.5 fs; equilibrium time: 1.0 ps; and simulation time: 1000, ps. The average energy between clockwise tilting and CCW tilting was compared with corroborate the preferred tilting direction triggered by the chiral oligoether dendron chains.

### Measurement of semipermeability of tubular wall

A series of guest molecules (Resorcinol, 4-methylumbelliferone, alizarin red S, cAMP, 8-hydroxypyrene-1,3,6-trisulfonic acid and calcein) were subjected to encapsulation and releasing experiment to evaluate the residue of the encapsulated guest molecules. Aqueous solution (1.0 ml) of guest molecules (2 mM) was added to 0.3 mg amphiphile **1** film to give 0.03 wt% amphiphile **1** aqueous solution. The mixture sample was sonicated for 20 min and stabilized for 3 h at room temperature. Then, the sample was aliquoted into two vials (each 0.5 ml). One vial was kept for 1 h at room temperature and the other vial was kept for 1 h at 45 °C. After 1 h, the two samples (0.2 ml) were subjected to Sephadex G-50 (700 mg) column to remove untrapped guest molecule. Eight fractions of 6.0 ml eluate (each 0.75 ml) were collected and monitored for the residue of guest molecules through HPLC analysis or UV/Vis measurement. In each fraction, absorbance in *λ*_max_ of guest molecules was normalized with concentration of amphiphile **1** measured from absorbance at 250 nm, providing the normalized residue of guest molecules in the tubules. From the normalized residue, the releasing percentage was plotted against molecular volume to show the size effect of permeable guest molecules.

### Releasing rate of the encapsulated calcein

Two independent experiments were performed to evaluate the releasing rate of the encapsulated calcein. One experiment was based on UV/Vis measurement. The procedure was followed as explained in the method of measurement of semipermeability. Total five aliquots (0.03 wt% amphiphile **1** in 2 mM calcein aqueous solution) were prepared and incubated at 45 °C for a period of time. After Sephadex-50 column purification, fractions of eluate were subjected to on UV/Vis measurement to provide the residue of calcein in tubules. Absorption spectra were normalized with concentration of amphiphile **1** measured from absorbance at 250 nm Finally, the releasing percentage of calcein from tubules was plotted against heating time. The other experiment was based on fluorescence quenching of calcein. Aqueous solution (1.0 ml) of calcein (100 mM) was added to 0.3 mg amphiphile **1** film to give 0.03 wt% amphiphile **1** aqueous solution. The mixture sample was sonicated for 20 min at room temperature. After stabilizing for 3 h at room temperature, untrapped free calcein was removed by filtration over a Sephadex G-50 column. The early fraction containing the tubules with calcein was collected and subjected to fluorescence measurements. The fluorescence intensity was measured with excitation at 491 nm and emission at 515 nm. For the releasing study of encapsulated calcein from closed tubule, the early fraction containing the tubules with calcein was incubated in 45-°C oven and the fluorescence intensity was also measured in 45 °C.

### Dehydration reaction from AMP to cAMP

Aqueous solution (1.0 ml) of AMP (10 mM) was added to 0.3 mg amphiphile **1** film to give 0.03 wt% amphiphile **1** aqueous solution. The mixture sample was sonicated for 20 min at room temperature. After stabilizing for 3 h at room temperature, the mixture (0.5 ml) was subjected to Sephadex G-50 column. Fourteen fractions of 10.5-ml eluate (each 0.75 ml) were collected and monitored for AMP content by measuring the absorbance intensity at 254 nm in analytical HPLC (eluent condition: 5% CH_3_CN in water with 0.1% trifluoroacetic acid). The early fraction containing the tubules with AMP was subjected to UV/Vis, fluorescence measurements, circular dichroism and dynamic light scattering measurement, and TEM experiments. To study the dehydration reaction using the close state of tubules, the encapsulated AMP was monitored by HPLC analysis in 45 °C for 12 h. The open–closed switching cycles of the tubular pores was performed with 0.03 wt% amphiphile **1** aqueous solution in the presence of free AMP (10 mM) without Sephadex separation. In each open–closed switching cycles, the solution was incubated in 45-°C oven for 5 h and cooled down to room temperature for 12 h with mild sonication. The resulting cAMP by the open–closed switching cycles was monitored by HPLC analysis (eluent condition: 5% CH_3_CN in water with 0.1% trifluoroacetic acid). The control experiment is performed to confirm whether amphiphile **1** suspension (above LCST) in water without further self-assembling into tubules can catalyse dehydration reactions or not. Aqueous solution (0.5 ml) of AMP (20 mM) preheated to 45 °C was added slowly to 0.06 wt% amphiphile **1** in 45-°C aqueous solution (0.5 ml). The mixture sample was incubated in 45-°C oven for 5 h and then subjected to HPLC analysis.

### Dehydrative imine formation reaction

An aqueous solution (0.25 ml) of *p*-hydroxybenzaldehyde (20 mM) and an aqueous solution (0.25 ml) of *m*-phenylenediamine (5 mM) were added to 0.1 wt% aqueous amphiphile **1** solution (0.5 ml) to give 0.05 wt% aqueous amphiphile **1** solution with 5 mM *p*-hydroxybenzaldehyde and 1.25 mM *m*-phenylenediamine. The mixture sample was sonicated for 10 min and kept for 3 h at room temperature. Then, the sample was incubated in 45-°C oven for 2 h and cooled down to room temperature for 3 h. After 20 times of open–closed switching cycles, suspended imine product was centrifuged, washed with dH_2_O and dried vacuum. The resulting solid product was subjected to fluorescenerce measurement and mass spectrum.

## Additional information

**How to cite this article:** Kim, Y. *et al*. Open–closed switching of synthetic tubular pores. *Nat. Commun.* 6:8650 doi: 10.1038/ncomms9650 (2015).

## Supplementary Material

Supplementary Information Supplementary Figures 1-22, Supplementary Methods and Supplementary References
