# Supplementary material for: Open–closed switching of synthetic tubular pores
Source: Nat Commun. 2015 Oct 12;6:8650. doi: 10.1038/ncomms9650 (PMC4633957; doi:10.1038/ncomms9650)
Supplement: Supplementary Information — Supplementary Figures 1-22, Supplementary Methods and Supplementary References [file ncomms9650-s1.pdf]

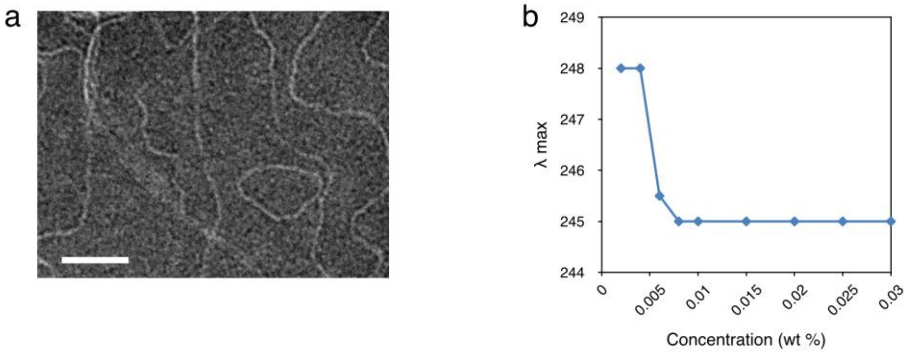

Supplementary Figure 1 Fibrils from low concentration of amphiphile 1 and critical concentration from fibrils to tubules. (**a**) Negative-stain TEM image of **1** from 0.003 wt % aqueous solution. The thin fibrils have an external diameter of ~4 nm, demonstrating that the tubular walls are composed of the lateral association of the primary fibrils formed at initial self-assembly conditions. Scale bar, 50 nm. (**b**) The wavelength of maximum absorption in different concentrations. The drastic change in 0.005 wt % suggests the transition between the fibrils and the tubes.

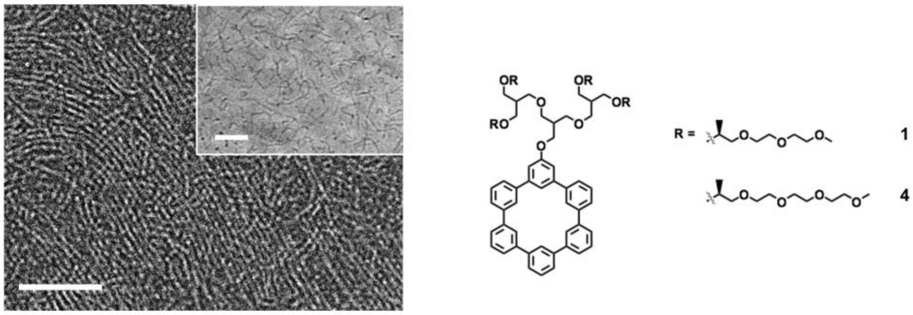

Supplementary Figure 2 Negative-stain TEM image and cryo-TEM image (inset) of 4 from 0.01wt % aqueous solution. The self-assembly of the macrocycle amphiphile **4** with longer hydrophilic chains showed the stable fibrils with ~5 nm in diameter. This fibrils did not aggregate into tubules even at higher concentrations. Scale bars, 50 nm.

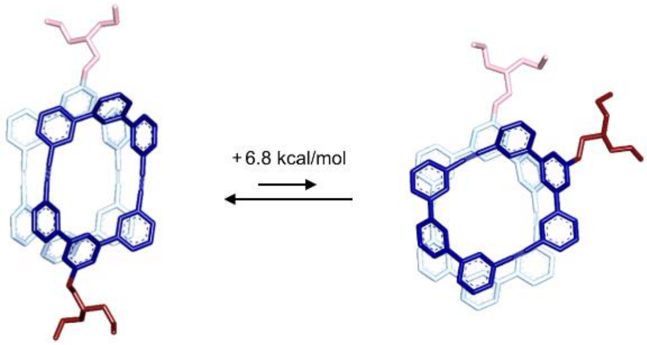

Supplementary Figure 3 Rotation energy calculation. The energy calculation of dimers of macrocycle amphiphile **2** based on an elliptical disc showed higher energy barrier in the mutual rotation of stacked aromatic discs than those of **1**.

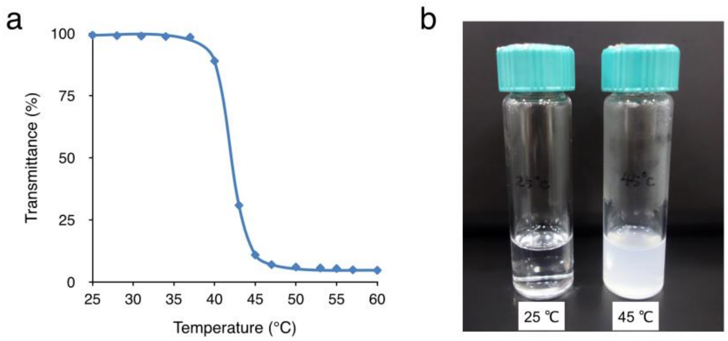

Supplementary Figure 4 Lower critical solution temperature (LCST). (**a**) The phase transition temperatures of the nanotubules in aqueous solution were obtained from the change in the transmittance. The transmittance at 500 nm was monitored with increase in the ambient temperature. The temperature-dependent transmittance of **1** from 0.03 wt % aqueous solutions showed a sharp phase transition at about 40 ^{o}C, indicating that the ethylene oxide chains in open conformations are dehydrated into a globular conformation upon heating. (**b**) Optical images of the solutions before and after heating shows the turbid sample solution over LCST. After the solution was placed in a 45 ^{o}C oven for one week, the image of the turbid solution was taken, suggesting the stable colloidal property of the tubules upon heating.

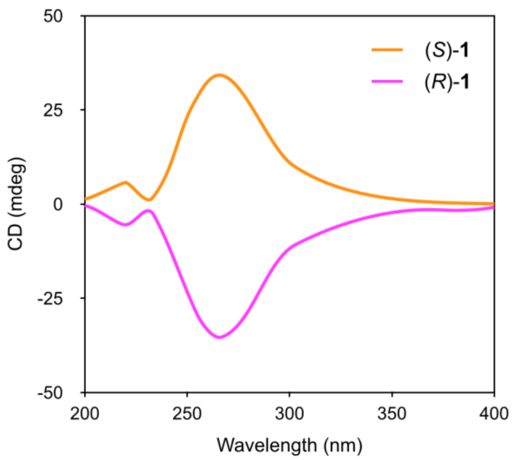

Supplementary Figure 5 Comparable CD spectra of (*S*)-1 and (*R*)-1 in 0.03 wt % aqueous solutions at 45 °C. (*R*)-**1** was synthesized according to the procedures reported in compound (*S*)-**1**. Assemblies derived from the enantiomer of (*S*)-**1** exhibit the opposite CD signal with a mirror image relationship, indicating that the molecular chirality of dendron is transferred to the self-assembled structures.

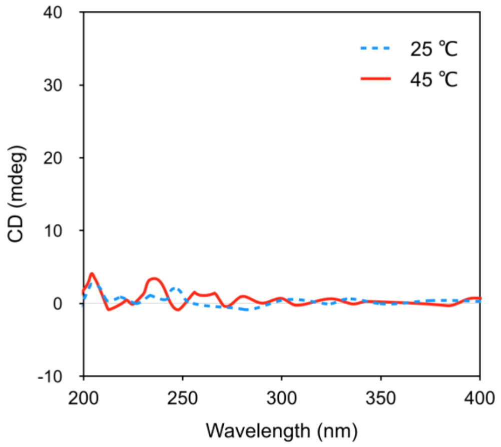

Supplementary Figure 6 CD suppression by amphiphile 3. CD spectra of 1 from 0.01 wt % aqueous solution in presence of 0.0005 wt % amphiphile 3 (inhibitor). A lack of CD signals suggests that tilting motion with a single direction cannot be induced by intercalation of inhibitor.

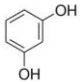

Resorcinol
M.W. 110.1
λ_{max} 270 nm

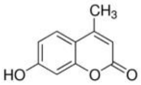

4-MU
M.W. 176.2
λ_{max} 323 nm

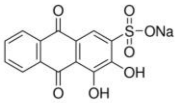

Alizarin Red S
M.W. 319.3
λ_{max} 488 nm

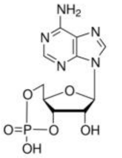

cAMP
M.W. 329.2
λ_{max} 260 nm

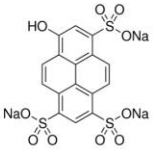

HPTS
M.W. 455.4
λ_{max} 406 nm

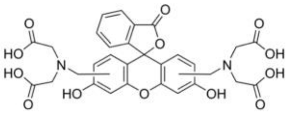

Calcein
M.W. 620.5
λ_{max} 491 nm

Guest	Formula	Vol (Å^3)	Releasing (%)
Resorcinol	$C_6H_6O_2$	104.1	**56.3**
4-MU	$C_{10}H_8O_3$	159.1	**33.1**
Alizarin Red S	$C_{14}H_7O_7S$	248.9	**48.4**
cAMP	$C_{10}H_{12}N_5O_6P$	250.0	**37.6**
HPTS	$C_{16}H_7O_{10}S_3$	331.1	**8.9**
Cacein	$C_{30}H_{24}N_2O_{13}$	534.1	**6.4**

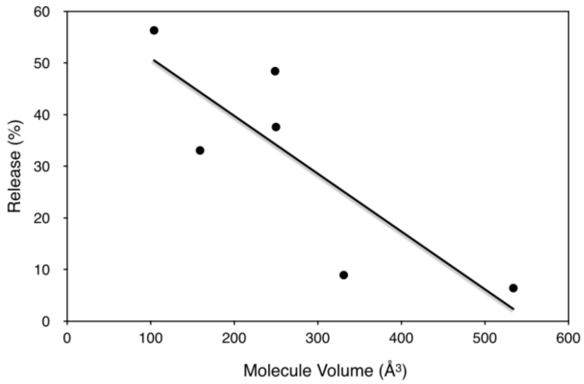

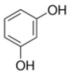

Resorcinol

Fraction	RT			LSCT		
	guest	amphiphile **1**	Ratio	guest	amphiphile **1**	Ratio
3	95.6	3171.5	0.03014	59.2	4352.1	0.0136
4	104.8	3261.5	0.03213			
average			0.03114			0.0136

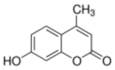

4-MU

Fraction	RT			LSCT		
	guest	amphiphile **1**	Ratio	guest	amphiphile **1**	Ratio
3	0.153	2.803	0.05458	0.081	2.217	0.03653

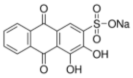

Alizarin Red S

Fraction	RT			LSCT		
	guest	amphiphile **1**	Ratio	guest	amphiphile **1**	Ratio
3	-	-	-	0.015	3.378	0.00444
4	0.023	2.672	0.00861			

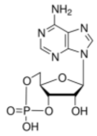

cAMP

Fraction	RT			LSCT		
	guest	amphiphile **1**	Ratio	guest	amphiphile **1**	Ratio
3	43.7	701.6	0.0623	113.1	3152.9	0.03587
4	382.2	4887.1	0.0782	155.1	2993.7	0.05181
average			0.07025			0.04384

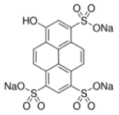

HPTS

Fraction	RT			LSCT		
	guest	amphiphile **1**	Ratio	guest	amphiphile **1**	Ratio
3	0.027	2.185	0.01236	0.021	1.617	0.01299
4	0.035	1.642	0.02132	0.043	2.432	0.01768
average			0.01684			0.01533

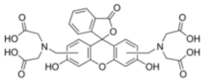

Calcein

Fraction	RT			LSCT		
	guest	amphiphile **1**	Ratio	guest	amphiphile **1**	Ratio
3	0.371	0.975	0.3805	0.218	0.639	0.3412
4	0.584	0.521	1.1215	0.418	0.392	1.0663
average			0.7510			0.7037

Supplementary Figure 7 Measurement of semipermeability of tubular wall. A series of guest molecules were encapsulated to tubular cavity and releasing amount of guest molecules after 1 hour incubation at 45 ^{o}C was measured. Absorbance in λ_{max} of guest molecules was normalized with concentration of amphiphile **1** measured from absorbance at 250 nm, indicating the normalized residue of guest molecules in tubules. Molecular volume (Å^3) was calculated from the VDW surface after geometry optimization through the COMPASS force field. From the normalized residue, the release percentage was plotted against molecular volume (Å^3), showing a linear relationship. Eventually the larger molecules are released out more slowly, demonstrating the semipermeable characteristics across the tubular wall.

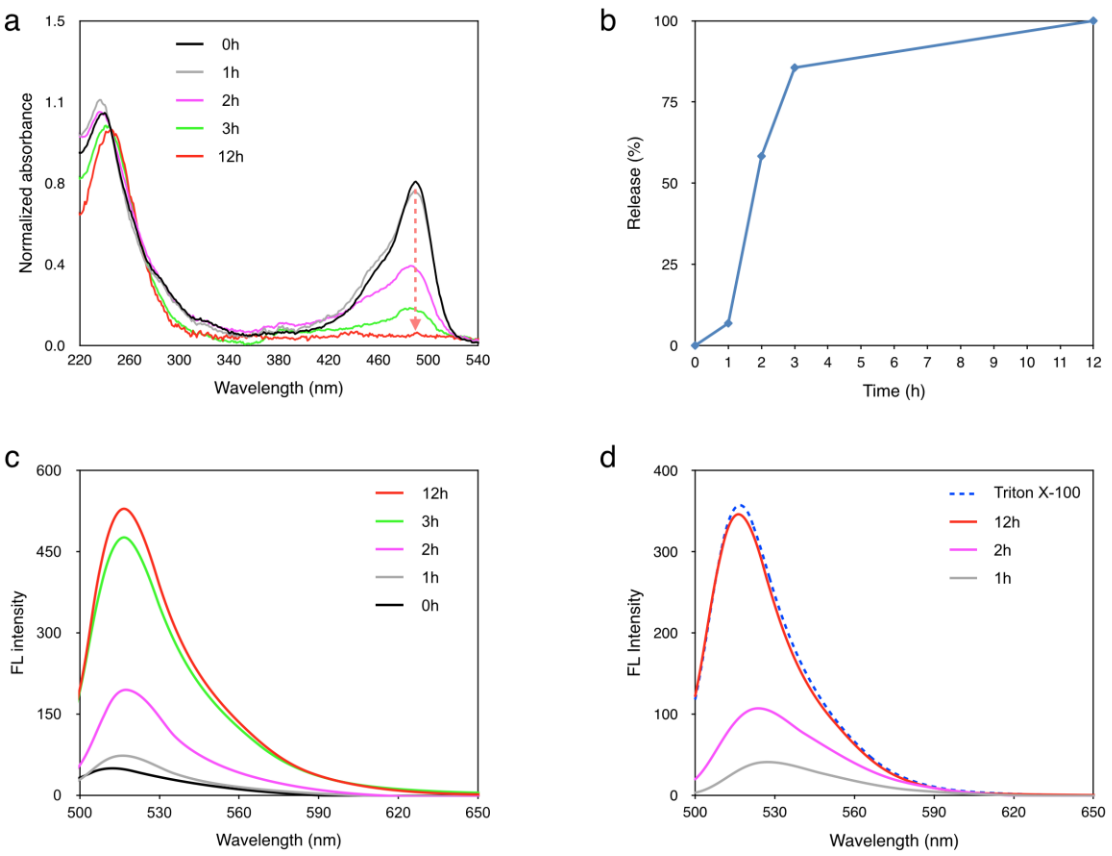

Supplementary Figure 8 Release rate of the encapsulated calcein. (**a**) Normalized absorption spectra from the eluate fraction of Sephadex-50 column after incubation at 45 ^{o}C for a period of time (0h, 1h, 2h, 3h, and 12h). (**b**) Release percentage from absorbance in 491 nm plotted against heating time. (**c**) Fluorescence emission spectra at 45 ^{o}C for a period of time (0h, 1h, 2h, 3h, and 12h). The increase of fluorescence intensity indicated the release of the encapsulated calcein. (**d**) Addition (10 μL) of Triton X-100 (1 wt % aqueous solution) after 12 hour incubation of calcein encapsulated sample solution indicated that encapsulated calcein dye molecules are completely released out within a period of 12 hours.

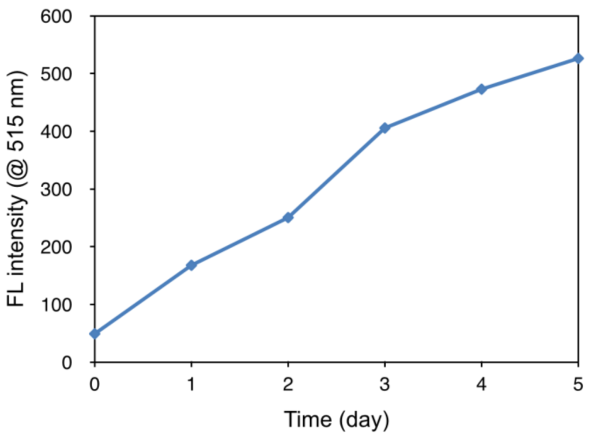

Supplementary Figure 9 Encapsulated calcein release experiment at room temperature. Variation in fluorescence emission spectrum with respect to time for calcein–loaded tubules at room temperature. Calcein was diffused out from the tubular pores over a period of 5 days in the open state at room temperature.

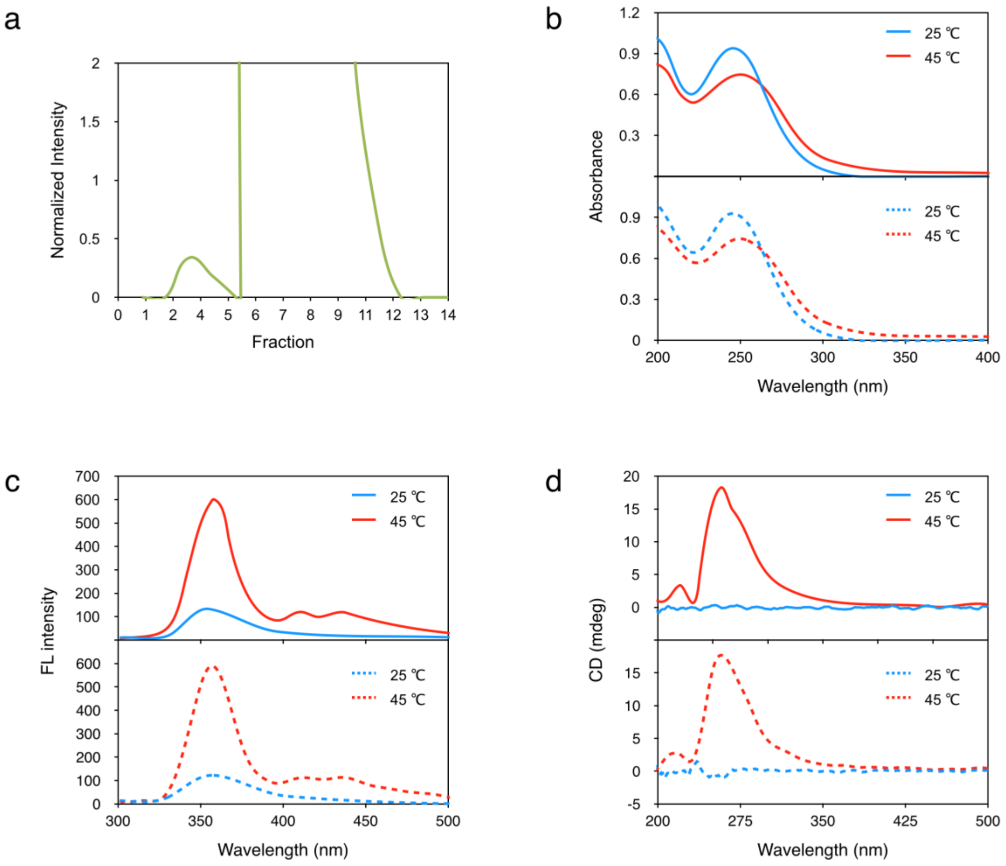

Supplementary Figure 10 Encapsulation of AMP and cAMP into tubular pores. (**a**) Profile of fraction of eluate from Sephadex-50 column. AMP content was monitored by measuring absorbance intensity at 254 nm in analytical HPLC. (**b**) Temperature-dependent absorption; (**c**) emission spectra; (**d**) CD spectra of **1** from 0.03 wt % aqueous solution with AMP encapsulation. The bottom spectra with dashed line were measured after the formation of cAMP upon heating. These results correspond to the spectroscopic data from pure aqueous solution **1** (0.03 wt %), indicating that AMP and cAMP do not interrupt the self-assembly packing of amphiphile **1**.

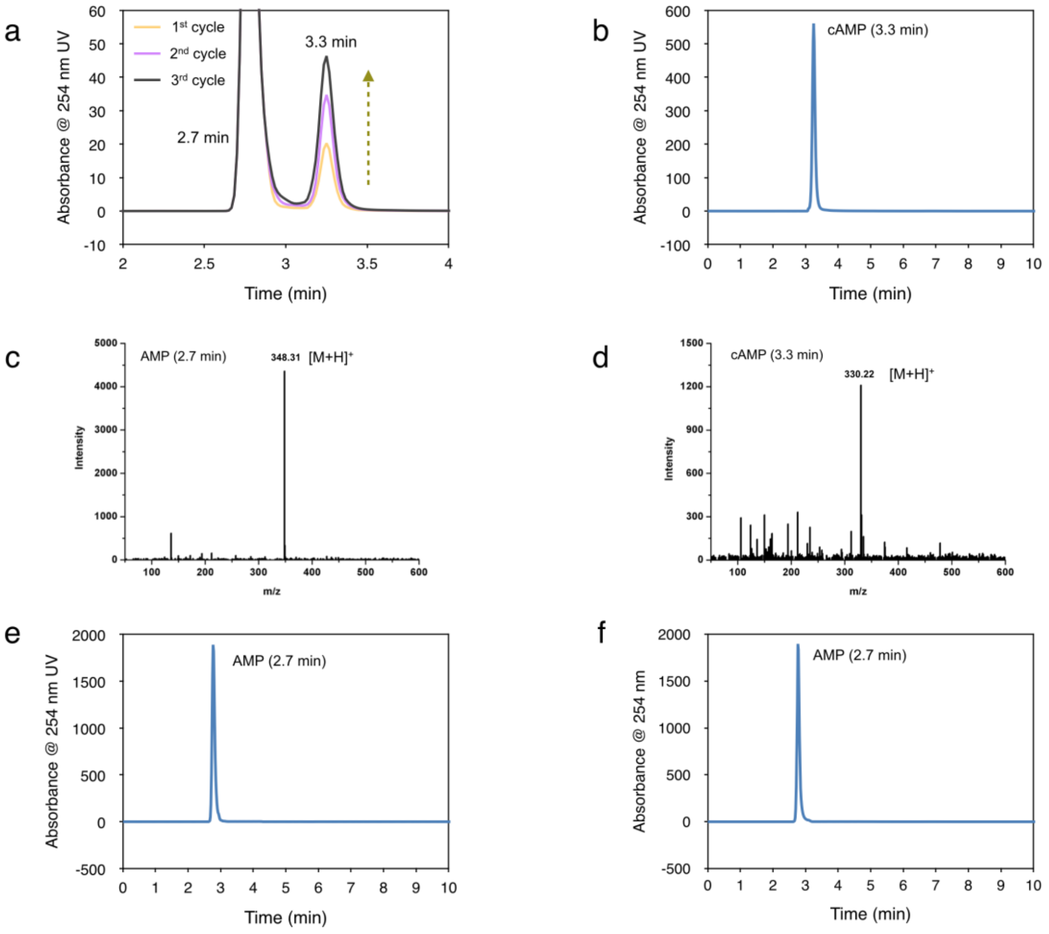

Supplementary Figure 11 Open-closed switching cycles of the tubular pores in dehydrative reaction for cAMP. (**a**) The open-closed switching cycles of the tubular pores with 0.03 wt % amphiphile **1** aqueous solution in the presence of free AMP (10 mM) without Sephadex separation. The peak intensity of cAMP through the open-closed switching cycles was increased during three cycles. (**b**) Commercial available pure cAMP molecule (TCI Product Number: A2381) was measured as a reference and it shows a peak in 3.3 min. (**c**) ESI-MS spectra of 2.7 min peak indicated AMP molecular mass and (**d**) ESI-MS spectra of 3.3 min peak indicated cAMP molecular mass (**e**) Blank experiment from 10 mM AMP aqueous solution without tubules at 45 ^{o}C for 12 hr. (**f**) The control experiment from amphiphile **1** suspension at 45 °C in 0.03 wt % **1** in 10 mM AMP solution without further self-assembling into tubules. This result showed that there was no dehydrative product of AMP, demonstrating that tubular structure play a vital role in dehydration reaction.

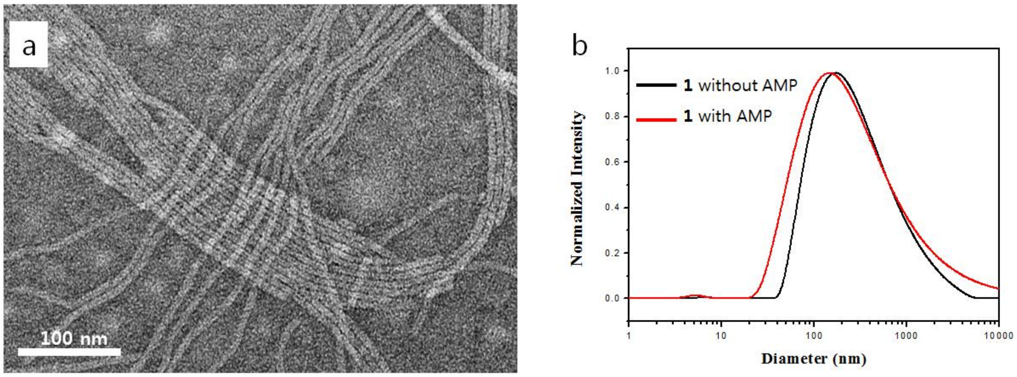

Supplementary Figure 12 Encapsulation of AMP into tubular pores. (**a**) Negative-stain TEM image of **1** from 0.01 wt % aqueous solution with AMP encapsulation. (**b**) Size distribution graph from DLS measurements. These data imply that AMP do not interrupt the self-assembly packing of amphiphile **1**.

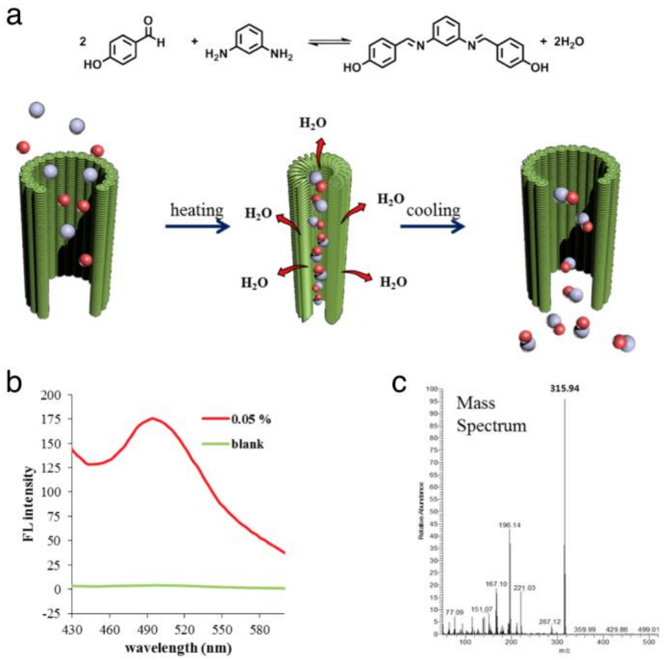

Supplementary Figure 13 Dehydrative imine formation reaction. Diimine formation from *p*-Hydroxybenzaldehyde and *m*-phenylenediamine was performed through water-pumping action of synthetic tubule. (**a**) Reaction scheme of dehydrative imine formation reaction and schematic representation of the dehydration reaction through water-pumping action of synthetic tubule. (**b**) Fluorescence emission spectra; (**c**) mass spectrum of diimine product from twenty water-pumping cycle.

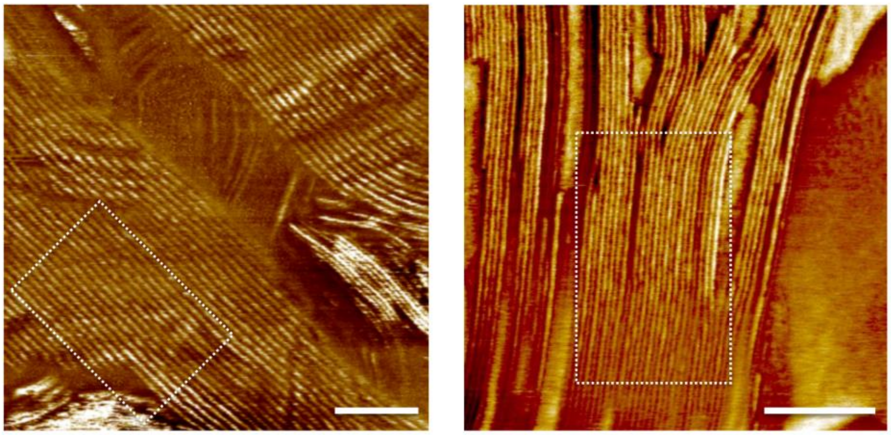

Supplementary Figure 14 AFM phase images. AFM phase images of the films on mica surface from evaporation of aqueous solution of **1** from 0.01 wt % aqueous solution prepared at (**a**) 25 °C and (**b**) 45 °C. To prepare the heated sample, 0.01 wt % aqueous solution was incubated in 45 °C oven for 1 h. Then, 10 μL aliquots of the turbid solution were cast on mica surface and the solvent was evaporated in 45 °C oven for 30 min. Magnified AFM phase images corresponding to the areas indicated by the white square are shown in Figure 3b. Scale bars, 100 nm.

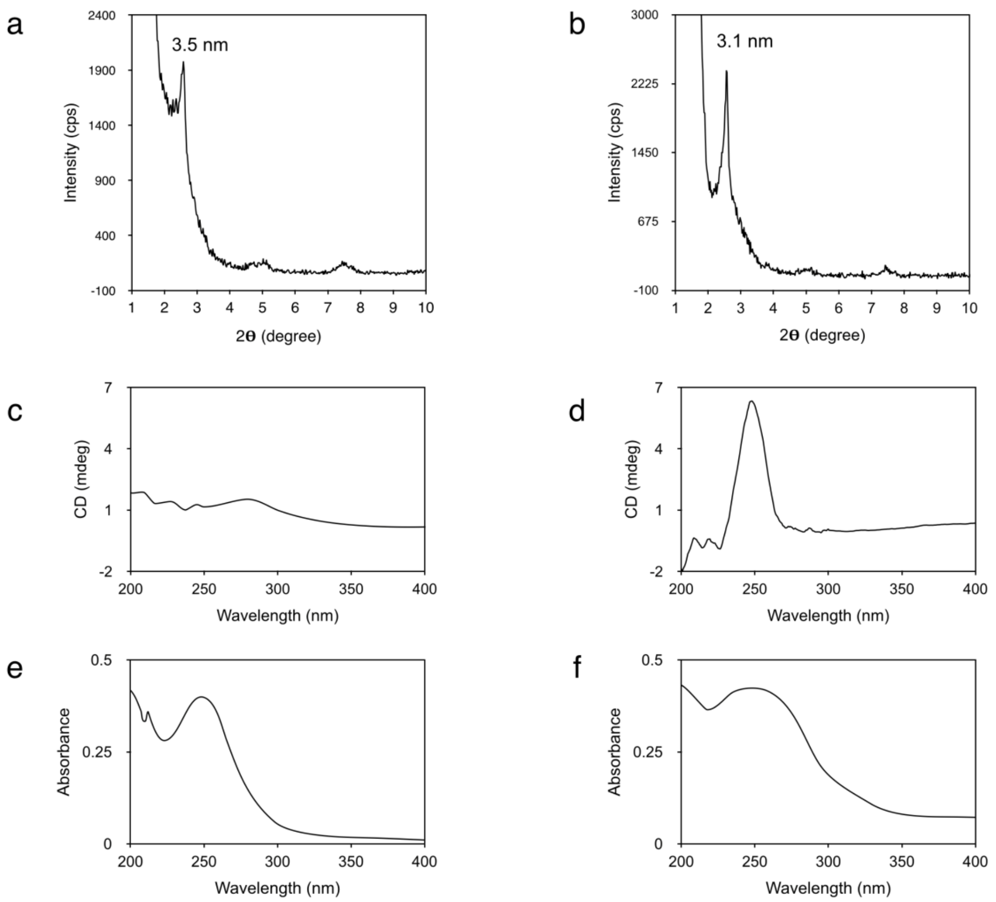

Supplementary Figure 15 XRD pattern, CD spectra, and absorption spectra. These data were collected from the films from evaporation of aqueous solution of **1** from 0.01wt % aqueous solution prepared at 25 °C (**a**, **c**, **e**) and 45 °C (**b**, **d**, **f**).

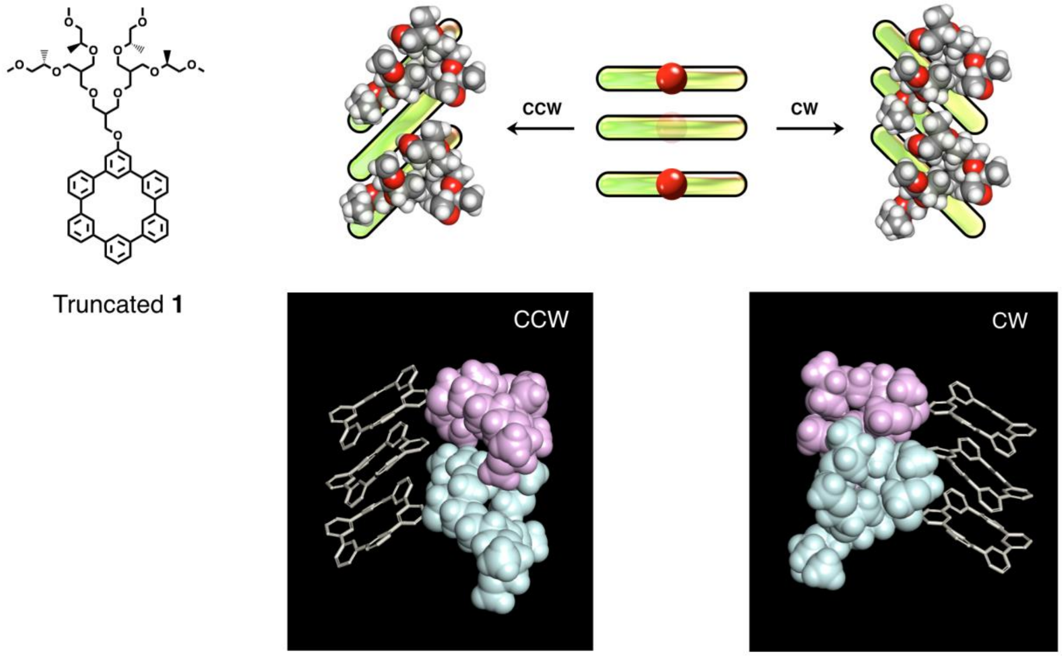

			CCW	CW
**Energy**	Average potential energy		1217.84 kJ/mol	1193.10 kJ/mol
	Average kinetic energy		1064.65 kJ/mol (Av temperature = 296.4 deg K)	1065.16 kJ/mol (Av temperature = 296.5 deg K)
	Average total energy		**2282.50 kJ/mol (Std dev = 77.30 kJ/mol)**	**2258.26 kJ/mol (Std dev = 77.02 kJ/mol)**
**Surface Area (SA) & Volume of Dendrimer Segment**	VDW approach	SA (Å²)	1361	1354
		Vol (Å³)	1172	1175
		SA / Vol ratio	**1.161**	**1.152**
	Solvent surface approach (Probe radius: 1.4)	SA (Å²)	988	898
		Vol (Å³)	1319	1355
		SA / Vol ratio	**0.749**	**0.663**

Supplementary Figure 16 Molecular dynamics simulation for tilting direction. Molecular dynamics simulation results of truncated **1** to compare the average energy for flexible oligoether chain conformation between clockwise (CW) tilting and counterclockwise (CCW) tilting. CW tilting allows the anisotropic chiral oligoether chains to adopt lower average energy and more compact packing compared with those of CCW tilting.

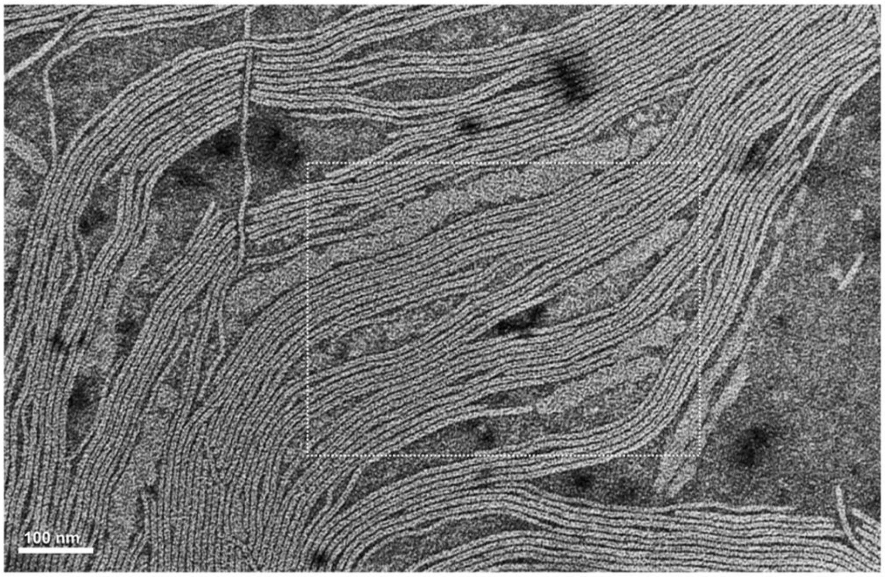

Supplementary Figure 17 The larger area of negative-stain TEM image of self-assembled tubules. The larger area of negative-stain (0.4 wt % uranyl acetate) TEM image from 0.01 wt % aqueous solution at room temperature. Magnified TEM image corresponding to the area indicated by the white square is shown in Figure 1c.

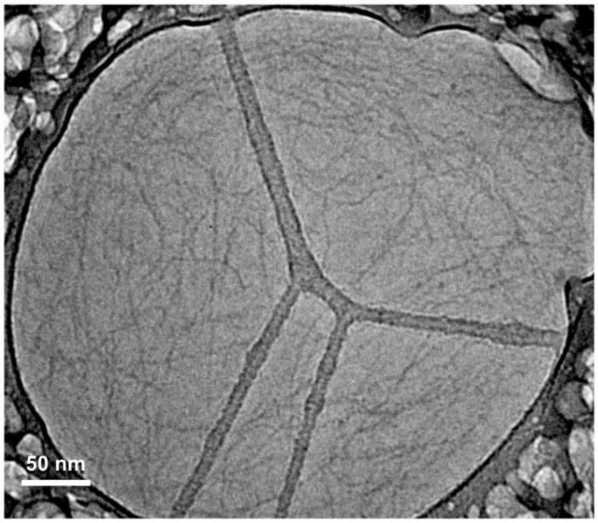

Supplementary Figure 18 The larger area of cryo-TEM image of closed tubules. The larger area of cryo-TEM image of **1** from 0.01wt % aqueous solution prepared at 45 °C.

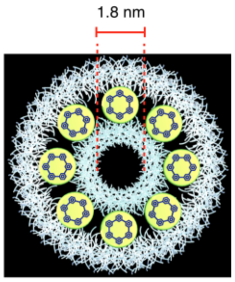

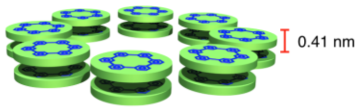

Unit Volume = $\pi \times (0.9)^2 \times 0.41 = 1.043$ (nm^3)

M.W. 1377.73	Weight Percent	Molarity	Number of **1**	Number of Unit	Total Tubular Pore Volume
Concentration of Amphiphile **1**	0.3 mg / 1 mL (0.03 wt %)	218 nmol / 1 mL = 218 uM	1.31×10^{17} / 1 mL	1.64×10^{16} / 1 mL	1.71×10^{16} (nm^3) = 1.71×10^{-5} (mL)
**Calculated volume ratio of tubular pore in 1 mL sample solution: 1.71×10^{-5}**					

From Supplementary Figure 10a	Encapsulated AMP (Fraction 2~5)	Untrapped AMP (Fraction 6~12)	Ratio of Encapsulation of AMP
Peak Area in Fraction Profile	1.048	9.878×10^4	1.061×10^{-5}
**Ratio of encapsulation of AMP from 0.03 wt % 1 in an aqueous AMP solution (10 mM): 1.061×10^{-5}**<br>**∴AMP concentration in tubular pore: 106.1 nM**			

From Figure 4b	AMP (RT 2.7 min)	cAMP (RT 3.3 min)	Reaction Yield (%) on the basis of encapsulated AMP
Peak Area (mV × s)	1071.6	119.6	10.04
**∴ Reaction yield in bulk sample solution per cycle = $1.061 \times 10^{-5} \times 10.04$ % = 1.065×10^{-4} %**<br>**∴ cAMP concentration per cycle = 10.65 nM**			

Supplementary Figure 19 Reaction yield in bulk sample solution per cycle. The stacking distance of cyclohexa-*m*-phenylene was reported as 0.41 nm[20]. The volume ratio of tubular pore compared to volume of aqueous solution was calculated as 1.71×10^{-5} from the tubular dimension, and observed as 1.061×10^{-5} from the peak area in fraction profile of AMP encapsulation (Supplementary Figure 10a). From the peak area of figure 4b, dehydration reaction yield on the basis of encapsulated AMP was observed as 10.04 %. Accordingly, the dehydration reaction yield of AMP in bulk sample solution per cycle was obtained as 1.065×10^{-4} %.

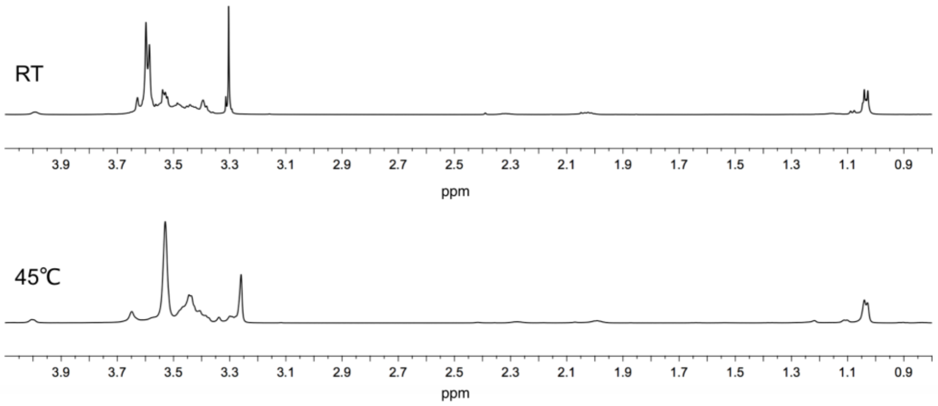

Supplementary Figure 20. NMR spectra for dehydration of dendron segment. ^{1}H-NMR (600 MHz) spectra were recorded using a solution of amphiphile **1** in D_2O with a concentration of 0.06 wt %. The figure shows that the resonances (0.9 ppm ~ 4.0 ppm) associated with the oligoether chains located at the tubular wall surfaces are upfield shifted together with peak broadening, indicating dehydration of the ethylene oxide segments[4].

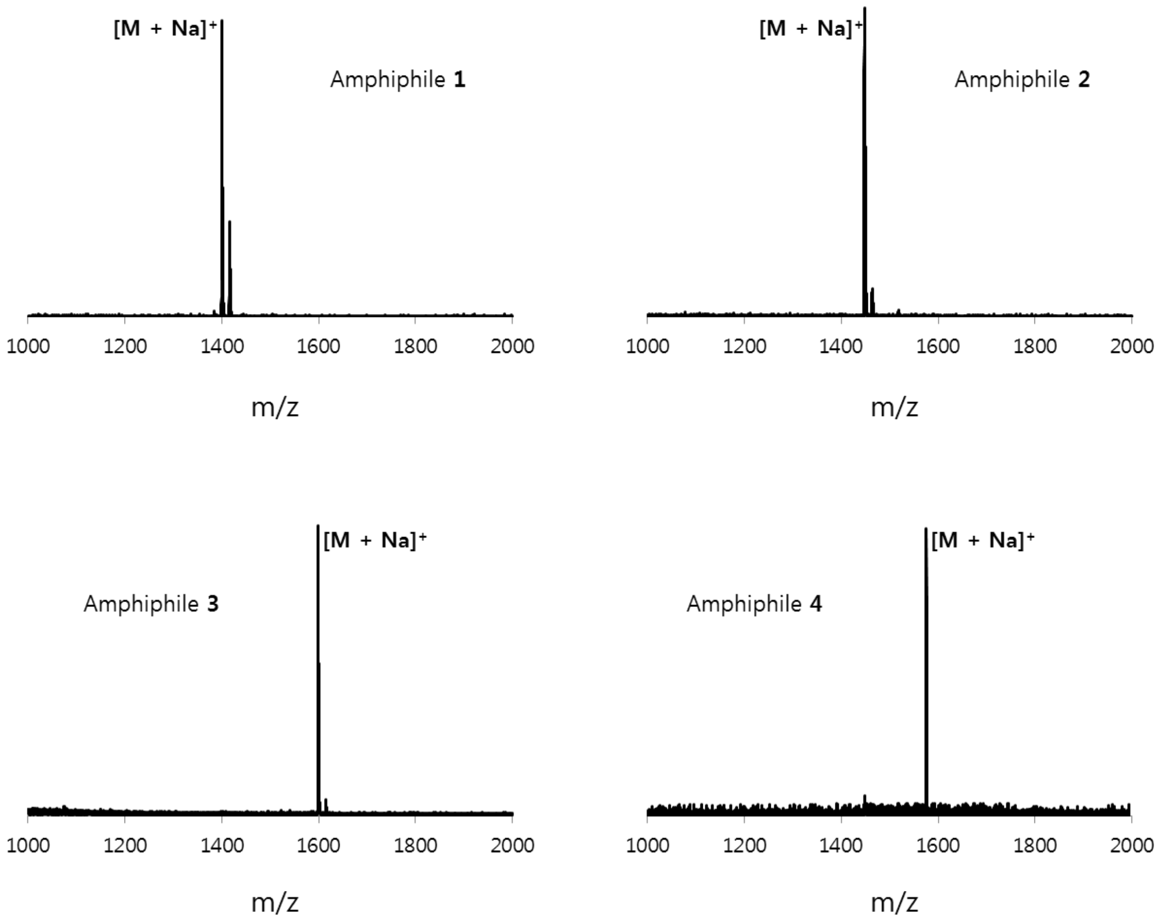

Supplementary Figure 21 MALDI-TOF mass spectrum of amphiphile 1–4.

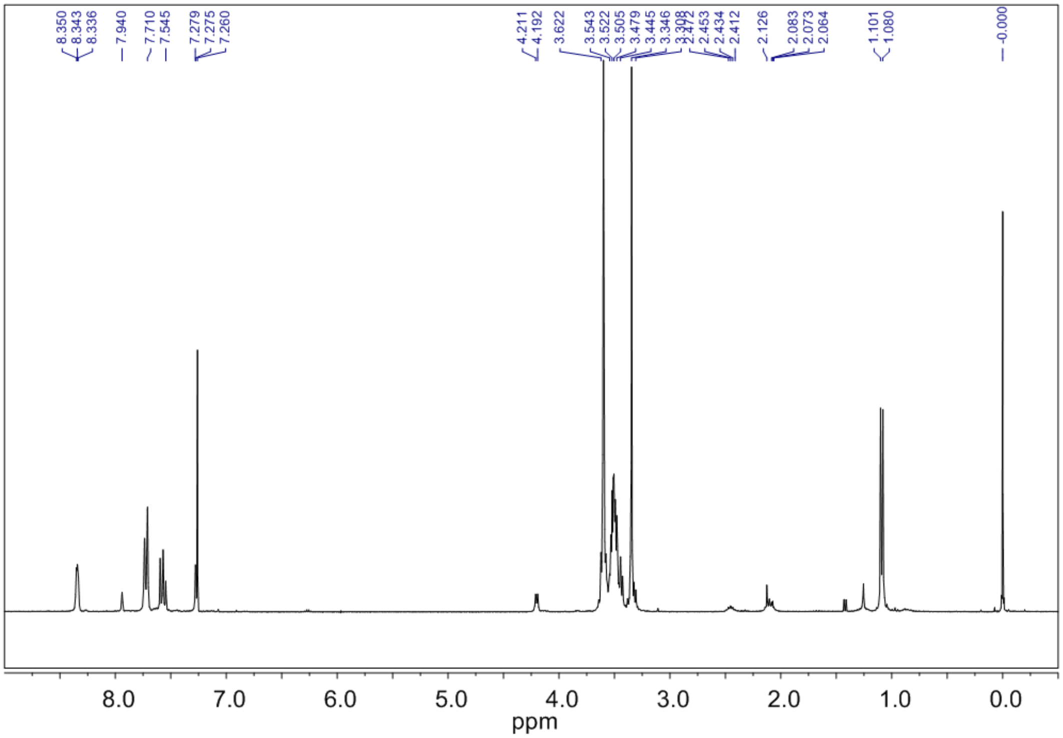

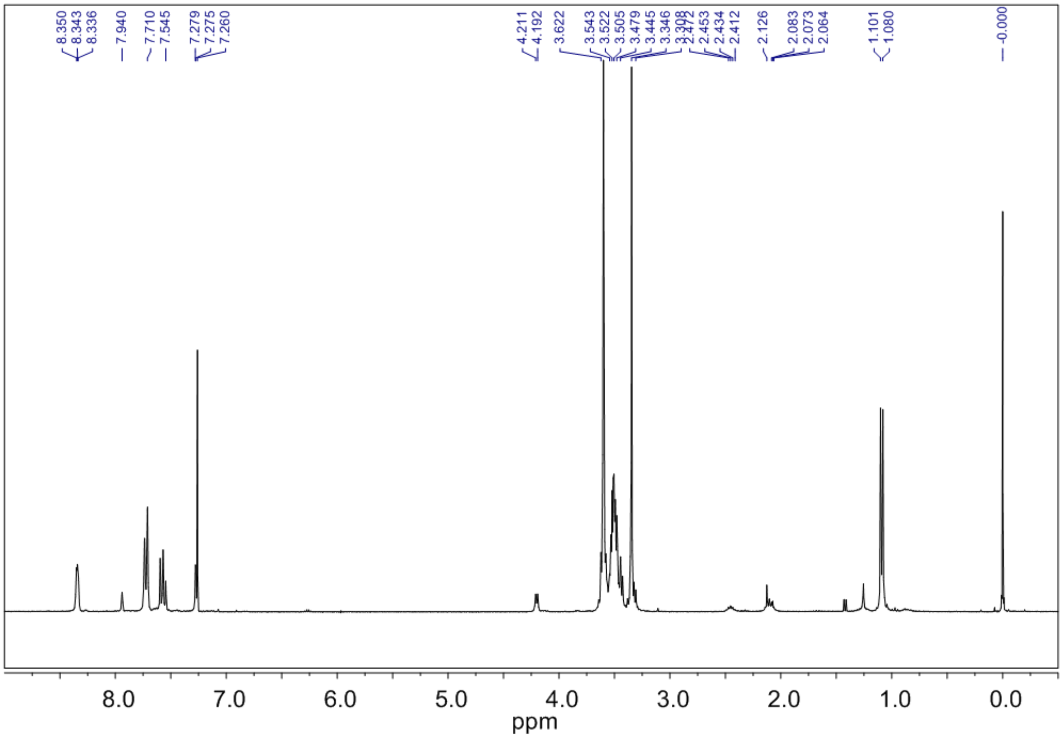

Supplementary Figure 22 NMR spectrum of amphiphile 1 in $CDCl_3$

Supplementary Methods

General Information

All reactions were performed either in oven-dried glassware under dry argon atmosphere. Toluene and tetrahydrofuran (THF) was dried by distillation from sodium–benzophenone immediately prior to use. Dichloromethane (DCM) was dried by distillation from CaH_2. Distilled water was polished by ion exchange and filtration. Other solvent and organic reagent were purchased from commercial vendors and used without further purification unless otherwise mentioned. The products were purified by flash column chromatography on silica gel (230–400 mesh). Thin-layer chromatography (TLC) was performed on precoated glass-backed plates (silica gel 60 F254 0.25 mm), and components were visualized by observation under UV light (254 and 365 nm) or by treating the plates with iodine, anisaldehyde, $KMnO_4$, phosphomolybdic acid, and vanillin followed by heating. ^{1}H and ^{13}C NMR spectra were obtained on 500 or 300 MHz FT-NMR spectrometer. All compounds are subjected to ^{1}H NMR analysis to confirm ≥98% sample purity. Chemical shifts were reported in ppm relative to the residual solvent peak ($CDCl_3$:^{1}H, 7.24; ^{13}C, 77.23) or tetramethylsilane (TMS) peak. Multiplicity was indicated as follows: s (singlet), d (doublet), t (triplet), q (quartet), quin (quintet), m (multiplet), dd (doublet of doublets), dt (doublet of triplets), td (triplet of doublets), br s (broad singlet). Coupling constants are reported in Hz. Recycling preparative high-pressure chromatography (HPLC) was performed for further purification by using HITACHI model pump L-7110, JAI model UV detector 310 and JAI model RI detector RI-7S. MALDI TOF-MS spectroscopy (MALDI-TOF-MS) was performed on a Bruker Autoflex TOF/TOF using α-cyano- 4-hydroxy cinnamic acid (CHCA) as a matrix. UV/Vis spectra were obtained from a Hitachi U-2900 Spectrophotometer. The fluorescence spectra were obtained from a FL-5301PC (SHIMADZU) fluorescence spectrophotometer. Circular Dichroism (CD) Spectra were obtained using Jasco J-810 spectropolarimeter. The AFM measurements were conducted on a MultiMode 8 AFM with NanoScope V controller, NanoScope software and NanoScope Analysis software (Bruker AXS Corporation, Santa Barbara, CA, USA) in air at ambient temperature (*ca*. 25°C) in the tapping mode. Images were acquired in Peak Force Tapping mode. X-ray diffraction (XRD) patterns were obtained using a Rigaku D/ max 2550 diffractometer (Rigaku Co.). HPLC analysis was performed with Prominence LC-20AP (SHIMADZU) and Vydac C18 reverse phase column (218TP54). Molecular modeling and calculations were performed by Material Studio 6.0 program (Accelrys Software Inc.) and the MacroModel module within the molecular modeling suite Maestro from Schrödinger Suites (Schrödinger K.K.).

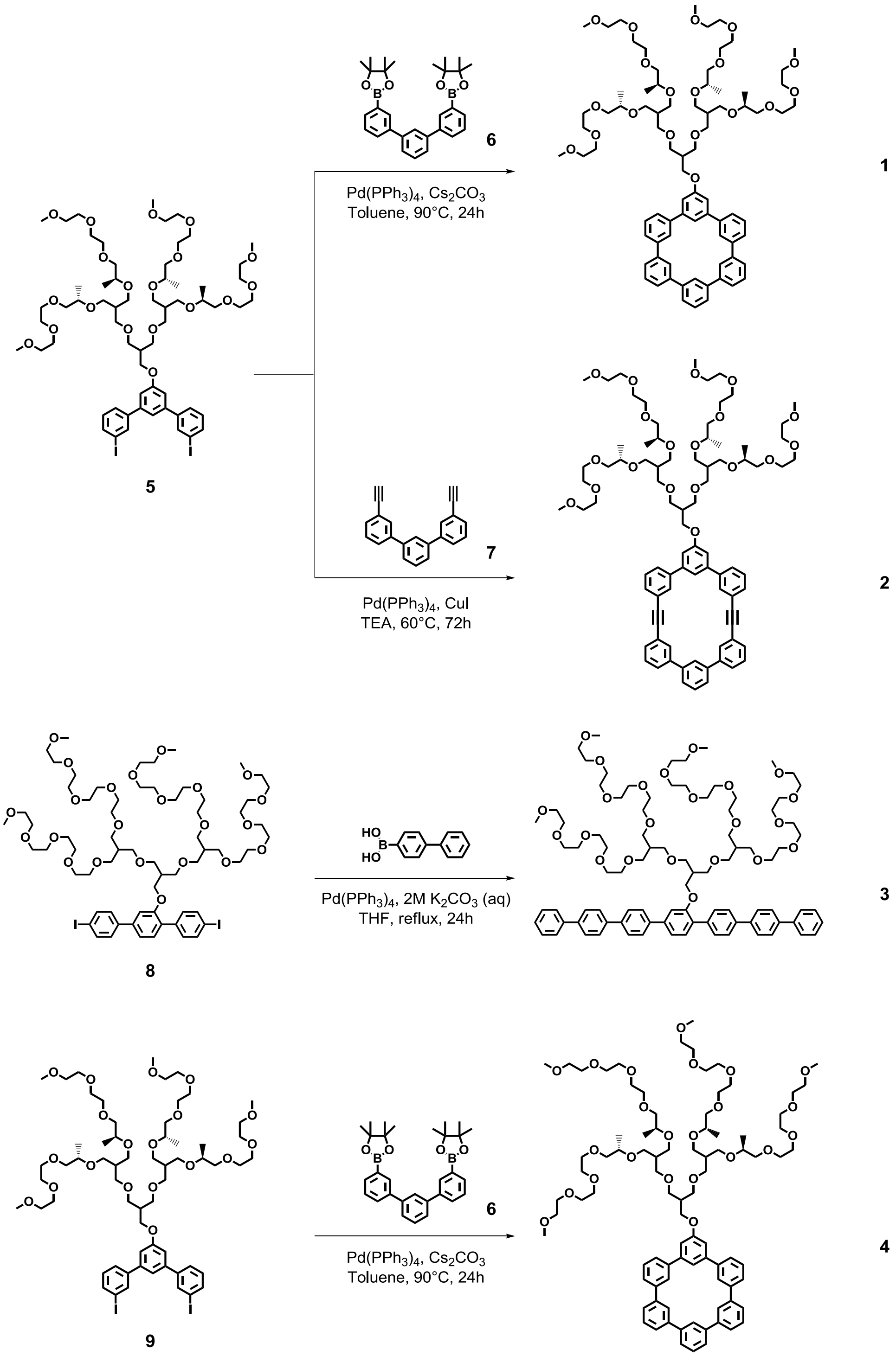

Synthetic method of macrocyclic amphiphiles and rod amphiphile.

Compound 1. Compound **5**[1] (118 mg, 0.084 mmol), compound **6** (41 mg, 0.084 mmol), and Cs_2CO_3 (274 mg, 0.84 mmol) were dissolved in degassed anhydrous toluene (16.8 mL). Then, $Pd(PPh_3)_4$ (1.9 mg, 0.00168 mmol) was added carefully. The mixture was stirred at 90 °C for 24 hours under atmospheric argon. After completion of the reaction as monitored by TLC, the reaction mixture was cooled down to room temperature and was diluted with ethyl acetate and washed with brine. The combined organic layer was dried over anhydrous $MgSO_4$(s). The filtrate was condensed under reduced pressure and purified by silica gel flash column using ethyl acetate / methanol (20:1 v/v) as eluent. Finally the purified product was subjected to further purification by JAI prep-HPLC to yield 18 % (18 mg) as a white solid.

^{1}H NMR (300 MHz, $CDCl_3$): δ 8.35–8.34 (m, 5H), 7.94 (s, 1H), 7.74–7.71 (m, 10H), 7.57 (t, *J* = 7.6 Hz 5H), 7.28 (d, *J* = 1.2 Hz, 2H), 4.20 (d, *J* = 5.7 Hz, 2H), 3.62–3.31 (m, 72H), 2.49–2.41 (m, 1H), 2.14–2.06 (m, 2H), 1.09 (d, *J* = 6.0 Hz, 12H); ^{13}C NMR (75 MHz, $CDCl_3$): δ 160.5, 142.6, 141.4, 141.4, 141.4, 141.3, 141.3, 129.7, 127.2, 127.1, 125.9, 125.8, 125.7, 125.7, 119.6, 119.2, 111.9, 99.9, 77.43, 77.0, 75.3, 75.0, 75.0, 72.2, 71.0, 70.8, 70.8, 70.7, 69.8, 69.6, 67.6, 66.6, 59.2, 41.1, 40.3, 29.8, 17.3; MALDI-TOF mass (m/z): $[M + Na]^+$ calcd. for $C_{80}H_{112}NaO_{19}$, 1399.77; found, 1399.46.

Compound 2. Compound **5**[1] (61 mg, 0.0435 mmol), compound **7** (12 mg, 0.0435 mmol), and CuI (8.3 mg, 0.0435 mmol) were dissolved in degassed anhydrous triethylamine (8.7 mL). Then, $Pd(PPh_3)_4$ (2.5 mg, 0.00218 mmol) was added carefully. The mixture was stirred at 60 °C for 72 hours under atmospheric argon. After completion of the reaction as monitored by TLC, the solvent was evaporated under reduced pressure. The resulting mixture was diluted with ethyl acetate and washed with brine. The combined organic layer was dried over anhydrous $MgSO_4$(s). The filtrate was condensed under reduced pressure and purified by silica gel flash column using ethyl acetate / methanol (20:1 v/v) as eluent. Finally the purified product was subjected to further purification by JAI prep-HPLC to yield 11 % (7 mg) as a white solid.

^{1}H NMR (500 MHz, $CDCl_3$): δ 8.16 (d, *J* = 8.1 Hz, 4H), 8.10 (s, 2H), 7.68 (t, *J* = 7.7 Hz, 6H), 7.58 – 7.55 (m, 4H), 7.50–7.46 (m, 5H), 7.20 (s, 2H), 4.16 (d, *J* = 5.1 Hz, 2H), 3.65–3.30 (m,

72H), 2.43–2.41 (m, 1H), 2.09–2.06 (m, 2H), 1.08 (d, J = 6.0 Hz, 12H); ^{13}C NMR (125 MHz, $CDCl_3$): δ 160.4, 142.0, 141.0, 141.0, 1410, 131.6, 131.7, 130.3, 130.2, 129.7, 127.0,126.9, 124.0, 124.0, 118.6, 112.3, 108.2, 107.9, 100.2, 77.43, 77.0, 75.5, 75.0, 75.0, 72.2, 71.0, 70.8, 70.8, 70.8, 69.8, 69.6, 67.7, 66.5, 59.2, 41.0, 40.3, 29.3, 17.3; MALDI-TOF mass (m/z): $[M + Na]^+$ calcd. for $C_{84}H_{112}NaO_{19}$, 1447.77; found, 1447.51.

Compound 3. Compound **8** was synthesized according to the procedures reported previously[2]. Compound **8** (50 mg, 0.0328 mmol) and biphenyl-4-boronic acid (33 mg, 0.164 mmol) were dissolved in degassed THF (0.7 mL). Degassed 2M aqueous K_2CO_3 (0.3 mL) was added to the resulting mixture and then, $Pd(PPh_3)_4$ (1.9 mg, 0.00164 mmol) was added carefully. The mixture was refluxed for 24 hours with vigorous stirring under atmospheric argon. After completion of the reaction as monitored by TLC, the reaction mixture was cooled down to room temperature and was extracted with ethyl acetate many times. The combined organic layer was dried over anhydrous $MgSO_4$(s). The filtrate was condensed under reduced pressure and purified by silica gel flash column using ethyl acetate / methanol (15:1 v/v) as eluent. Finally the purified product was subjected to further purification by JAI prep-HPLC to yield 67 % (35 mg) as a white solid.

^{1}H NMR (500 MHz, $CDCl_3$): δ 7.80–7.32 (m, 29H), 4.18 (d, J = 4.9 Hz, 2H), 3.64–3.38 (m, 92H), 2.42–2.40 (m, 1H), 2.07–2.03 (m, 2H); ^{13}C NMR (125 MHz, $CDCl_3$): δ 156.7, 141.0, 141.0, 140.9, 140.6, 140.5, 140.1, 140.1, 131.3, 130.3, 130.1, 127.8, 127.8, 127.7, 127.6, 127.3, 127.3, 126.7, 120.0, 120.0, 102.3, 77.44, 77.0, 75.1, 72.2, 70.8, 70.7, 70.7, 70.0, 69.8, 69.6, 59.3, 40.4, 40.2, 29.9; MALDI-TOF mass (m/z): $[M + Na]^+$ calcd. for $C_{90}H_{126}NaO_{23}$, 1597.86; found, 1597.59.

Compound 4. Compound **9**[3] (91 mg, 0.065 mmol), compound **6** (31 mg, 0.065 mmol), and Cs_2CO_3 (212 mg, 0.65 mmol) were dissolved in degassed anhydrous toluene (13 mL). Then, $Pd(PPh_3)_4$ (1.5 mg, 0.0013 mmol) was added carefully. The mixture was stirred at 90 °C for 24 hours under atmospheric argon. After completion of the reaction as monitored by TLC, the reaction mixture was cooled down to room temperature and was diluted with ethyl acetate and

washed with brine. The combined organic layer was dried over anhydrous $MgSO_4$(s). The filtrate was condensed under reduced pressure and purified by silica gel flash column using ethyl acetate / methanol (15:1 v/v) as eluent. Finally the purified product was subjected to further purification by JAI prep-HPLC to yield 15 % (15 mg) as a white solid.

^{1}H NMR (300 MHz, $CDCl_3$): δ 8.35–8.34 (m, 5H), 7.94 (s, 1H), 7.74–7.72 (m, 10H), 7.57 (t, *J* = 7.6 Hz 5H), 7.28 (d, *J* = 1.2 Hz, 2H), 4.20 (d, *J* = 5.7 Hz, 2H), 3.62–3.30 (m, 88H), 2.49–2.41 (m, 1H), 2.14–2.06 (m, 2H), 1.09 (d, *J* = 6.0 Hz, 12H); ^{13}C NMR (75 MHz, $CDCl_3$): δ 160.5, 142.6, 141.4, 141.4, 141.3, 141.3, 129.7 127.2, 127.1, 125.9, 125.8, 119.6, 111.9, 100.2, 77.4, 75.2, 75.0, 72.1, 70.9, 70.8, 70.7, 70.7, 69.8, 69.6, 67.6, 66.5, 59.2, 41.0, 40.3, 29.9, 17.3; MALDI-TOF mass (m/z): $[M + Na]^+$ calcd. for $C_{88}H_{128}NaO_{23}$, 1575.87; found, 1575.62.

Supplementary References

1. Kim, J.-K. *et al*. Reversible transformation of helical coils and straight rods in cylindrical assembly of elliptical macrocycles. *J. Am. Chem. Soc*. **131**, 17768-17770 (2009).

2. Lee, D.-W., Kim, T. & Lee, M. Amphiphilic pyrene sheet for selective functionalization of graphene. *Chem. Commun.* **47**, 8259-8261 (2011).

3. Huang, Z., Kim, Y., Kim, T. & Lee, M. Supramolecular polymerization of spherical micelles triggered by donor-acceptor interactions. *Polym. Chem.* **4**, 268-271 (2013).

4. Huang, Z. *et al*. Pulsating tubules from noncovalent macrocycles. *Science* **337**, 1521–1526 (2012).
